# Green multicomponent synthesis, antimicrobial and antioxidant evaluation of novel 5-amino-isoxazole-4-carbonitriles

**DOI:** 10.1186/s13065-018-0488-0

**Published:** 2018-11-15

**Authors:** Hamid Beyzaei, Mahboubeh Kamali Deljoo, Reza Aryan, Behzad Ghasemi, Mohammad Mehdi Zahedi, Mohammadreza Moghaddam-Manesh

**Affiliations:** 1 Department of Chemistry, Faculty of Science, University of Zabol, Zabol, Iran; 2 Torbat Jam Faculty of Medical Sciences, Torbat Jam, Iran; 3 Department of Chemistry, University of Saskatchewan, 110 Science Place, Saskatoon, SK S7N 5C9 Canada; 4 Young Researchers and Elite Club, Kerman Branch, Islamic Azad University, Kerman, Iran

**Keywords:** Antibacterial activity, Antifungal property, Antioxidant effect, Isoxazole, Multicomponent synthesis

## Abstract

**Background:**

Design and synthesis of new inhibitor agents to deal with pathogenic microorganisms is expanding. In this project, an efficient, environmentally friendly, economical, rapid and mild procedure was developed for the synthesis of novel functionalized isoxazole derivatives as antimicrobial potentials.

**Methods:**

Multicomponent reaction between malononitrile (**1**), hydroxylamine hydrochloride (**2**) and different aryl or heteroaryl aldehydes **3a**–**i** afforded novel 5-amino-isoxazole-4-carbonitriles **4a**–**i** in good product yields and short reaction times. Deep eutectic solvent K_2_CO_3_/glycerol was used as catalytic reaction media. Structure of all molecules were characterized by different analytical tools. In vitro inhibitory activity of all derivatives was evaluated against a variety of pathogenic bacteria including both Gram-negative and Gram-positive strains as well as some fungi. In addition, their free radical scavenging activities were assessed against DPPH.

**Results:**

Broad-spectrum antimicrobial activities were observed with isoxazoles **4a**, **b**, **d**. In addition, antioxidant activity of isoxazole **4i** was proven on DPPH.

**Conclusions:**

In this project, compounds **4a**, **b**, **d** could efficiently inhibit the growth of various bacterial and fungal pathogens. Antioxidant properties of derivative **4i** were also significant. These biologically active compounds are suitable candidates to synthesize new prodrugs and drugs due to the presence of different functional groups on their rings. 
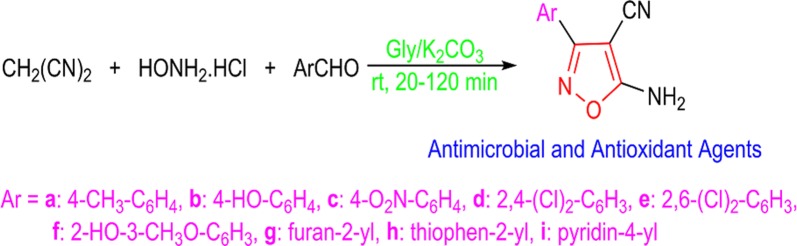

**Electronic supplementary material:**

The online version of this article (10.1186/s13065-018-0488-0) contains supplementary material, which is available to authorized users.

## Background

Isoxazoles are five-membered aromatic heterocycles containing adjacent oxygen and nitrogen atoms. The isoxazole ring system is found in a variety of naturally occurring compounds and biologically active molecules [1]. They are especially useful in medicine, since many antifungal drugs belong to the isoxazole class [2]. Sulfisoxazole and sulfamethoxazole are two bacteriostatic sulfonamide antibiotics that applied alone or combined with others in the treatment of infections caused Gram-positive and Gram-negative bacteria [3, 4]. Acivicin is a *γ*-glutamyl transferase inhibitor with anticancer, anti-parasitic and antileishmania activities [5]. Isoxazole derivatives possess a broad variety of biological activities viz. antifungal, anti-inflammatory, antiplatelet, anti-HIV, anti-Alzheimer and analgesic [6–11].

Cycloisomerization of α,β-acetylenic oximes [12], cycloaddition of aldoxime and alkynes [13], reaction of alkyl nitriles and *α*-chlorooximes [14], 1,3-dipolar cycloaddition of in situ generated nitrile oxides and terminal acetylenes [15, 16], addition of hydroxylamine to *α*-cyano ketones [17] and palladium-catalyzed four-component coupling of a terminal alkyne, hydroxylamine and carbon monoxide [18] are some recently developed methods for isoxazole synthesis. Furthermore, multicomponent reaction of active methylene compounds, aldehydes and hydroxylamine derivatives were well studied under different conditions [19–23].

Deep eutectic solvents (DES) play an essential key in green chemistry. They can be used as safe, low-cost, non-toxic, reusable, catalytic and environmentally friendly media in the most reactions [24]. Their applications are expanding in the field of materials, energy and environmental science [25]. Glycerol is a valuable green, nontoxic, low flammable and available solvent that applied as anti-freezer, sweetener, humectant, lubricant and thickener in industry [26]. This natural polyol as hydrogen bond donor is present in DESs with hydrogen bond acceptors such as choline chloride, methyl triphenyl phosphonium bromide, benzyl triphenyl phosphonium chloride, allyl triphenyl phosphonium bromide, *N*,*N*-diethylethanolammonium chloride, and tetra-*n*-butylammonium bromide [27]. Glycerol/potassium carbonate is a low cost and environmentally friendly DES that recently its efficiently was proven in the preparation of pyrazole derivatives [28].

**Fig. 1:**
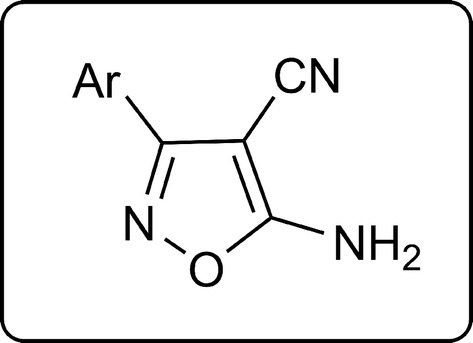
Schematic representation of isoxazole skeletons with antimicrobial and antioxidant activity

In order to develop applications of Gly/K_2_CO_3_ to other heterocycles, it was successfully used as catalytic media in the synthesis of novel 5-amino-isoxazole-4-carbonitrile derivatives via multicomponent reaction of malononitrile, hydroxylamine and various aryl aldehydes (Fig. 1). In vitro inhibitory activity of all derivatives was evaluated against some pathogenic bacteria including both Gram-negative and Gram-positive strains as well as some fungi. In addition, their antioxidant potentials were assessed against DPPH.

## Results

### Characterization of isoxazoles **4a**–**i**

**Scheme 1:**
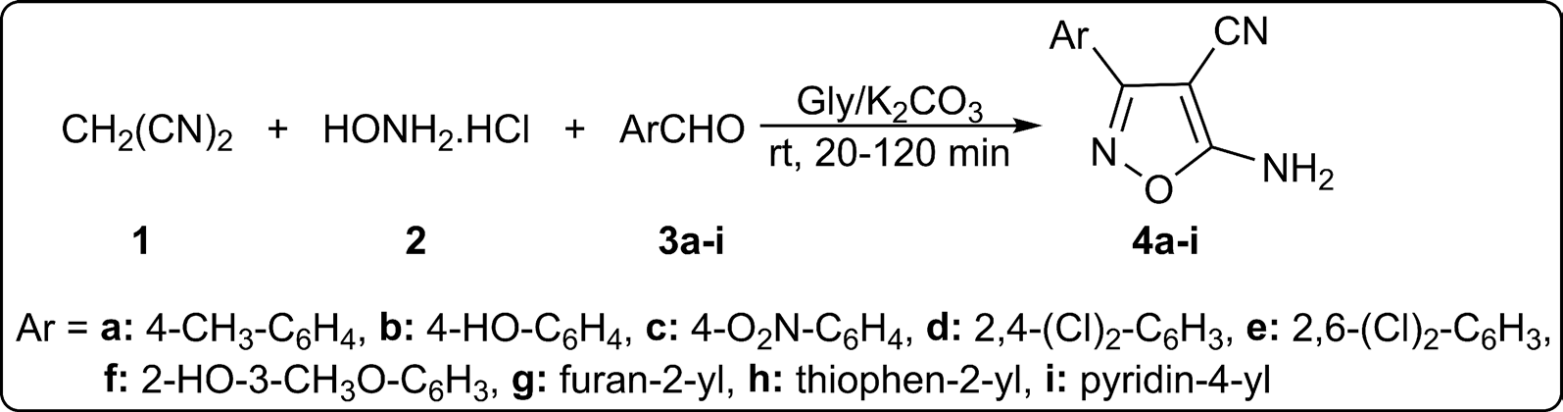
Multicomponent synthesis of 5-amino-isoxazole-4-carbonitriles

Multicomponent reaction of malononitrile (**1**), hydroxylamine hydrochloride (**2**) and aryl or heteroaryl aldehydes **3a**–**i** afforded 5-amino-isoxazole-4-carbonitriles **4a**–**i** in 70–94% yields (Scheme 1). Products were obtained in glycerol/potassium carbonate (4:1) at room temperature for 20–120 min.

### Evaluation of the bioactivity of isoxazoles **4a**–**i**

All synthesized compounds were assessed for their antimicrobial efficiency as well as antioxidant activity. Inhibitory effects of isoxazoles **4a**–**i** were presented as MIC, MBC and MFC values in Tables 1 and 2.

**Table 1:** Antibacterial effects of isoxazoles **4a**–**i**

Bacterial species		Products									Antibiotic
		4a	4b	4c	4d	4e	4f	4g	4h	4i	Gentamicin
1310	MIC	256	128	–	–	256	–	–	–	–	0.063
	MBC	512	256	–	–	512	–	–	–	–	0.063
1290	MIC	64	256	–	–	256	–	–	–	–	4
	MBC	128	512	–	–	512	–	–	–	–	4
1234	MIC	–	32	–	–	–	–	–	–	–	2
	MBC	–	64	–	–	–	–	–	–	–	8
1188	MIC	–	–	–	128	–	–	–	–	–	0.031
	MBC	–	–	–	512	–	–	–	–	–	0.063
1855	MIC	–	512	–	128	–	–	–	–	–	16
	MBC	–	2048	–	256	–	–	–	–	–	32
1399	MIC	–	–	–	32	–	–	–	–	–	8
	MBC	–	–	–	128	–	–	–	–	–	8
1768	MIC	512	128	256	256	–	64	–	–	128	0.5
	MBC	1024	256	1024	512	–	128	–	–	256	1
1297	MIC	32	64	32	64	32	32	256	32	64	2
	MBC	128	128	64	128	128	64	512	64	128	2
1445	MIC	–	256	–	64	–	–	–	–	256	2
	MBC	–	512	–	128	–	–	–	–	512	2
1240	MIC	256	512	–	–	–	–	–	–	–	1
	MBC	512	1024	–	–	–	–	–	–	–	1
1633	MIC	–	64	–	256	256	–	–	–	–	2
	MBC	–	128	–	512	512	–	–	–	–	2
1023	MIC	128	–	–	–	256	–	–	–	–	0.063
	MBC	512	–	–	–	512	–	–	–	–	0.063
1435	MIC	–	–	–	–	64	–	–	–	128	1
	MBC	–	–	–	–	128	–	–	–	512	2
1494	MIC	32	–	–	128	–	64	–	–	–	1
	MBC	64	–	–	512	–	128	–	–	–	1
1189	MIC	128	256	–	–	256	–	512	–	–	1
	MBC	512	1024	–	–	512	–	1024	–	–	1
1665	MIC	256	64	–	64	128	–	128	–	–	0.25
	MBC	512	64	–	128	512	–	256	–	–	4
1447	MIC	64	32	–	256	128	512	–	–	–	0.063
	MBC	128	32	–	512	512	512	–	–	–	0.125

–: No noticeable antibacterial effect at concentration of 10,240 μg ml^−1^, MIC (μg ml^−1^), MBC (μg ml^−1^)

**Table 2:** Antifungal effects of isoxazoles **4a**–**i**

Fungal species		Products									Antifungal
		4a	4b	4c	4d	4e	4f	4g	4h	4i	Canazole
5027	MIC	–	128	–	64	–	–	–	–	–	256
	MFC	–	256	–	128	–	–	–	–	–	512
5115	MIC	64	256	–	128	–	–	–	–	–	256
	MFC	128	512	–	256	–	–	–	–	–	512
5009	MIC	128	64	–	256	–	–	–	–	–	32
	MFC	512	256	–	512	–	–	–	–	–	32

–: No noticeable antifungal effect at concentration of 10,240 μg ml^−1^, MIC (μg ml^−1^), MFC (μg ml^−1^)

## Discussion

### Chemistry

**Scheme 2:**
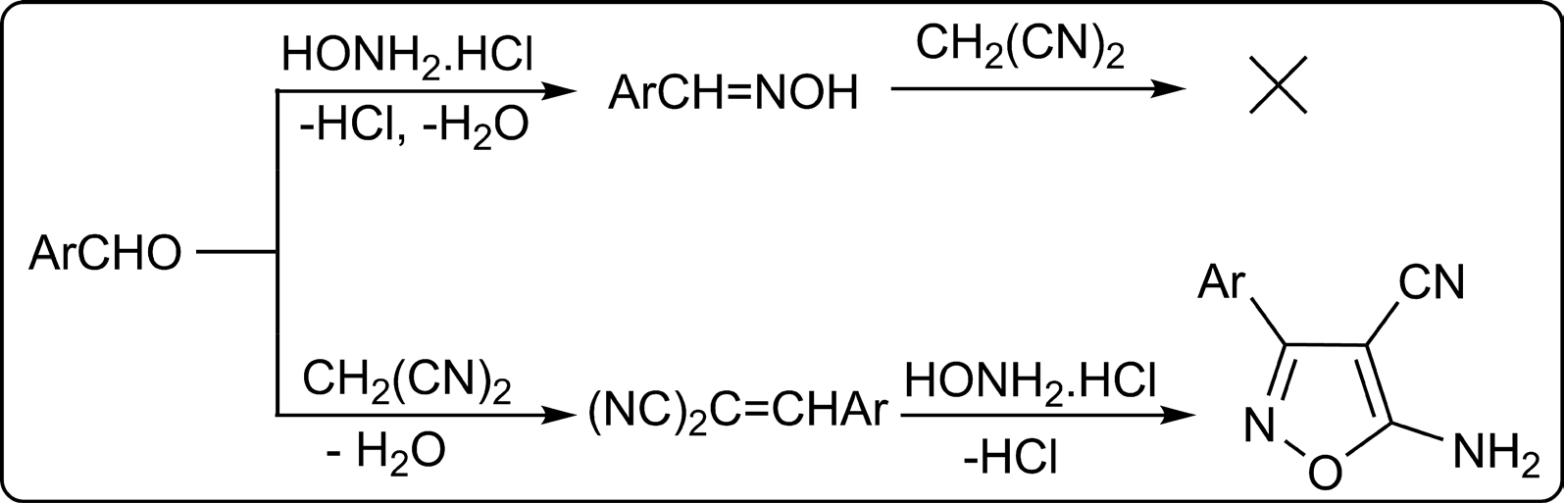
Proposed mechanisms for the formation of isoxazoles **4a**–**i**

The effects of variations in solvent, temperature and order mixing reactants were studied on product yield and reaction time. Aldoximes were produced as major products in glycerol at different conditions. They were also formed in Gly/K_2_CO_3_ deep eutectic solvents under one-pot two-step procedures involving initial mixing hydroxylamine and aldehydes, followed by malononitrile. In addition, oximes were present as by-products in one-pot two-step processes involving initial mixing malononitrile and aldehydes. There are two possible mechanisms to form the products (Scheme 2). A reaction pathway, that does not lead to the target products, includes the reaction of aldoximes produced from aldehydes and hydroxylamine with malononitrile. On another path, the Knoevenagel condensation of aldehydes with malononitrile gives arylidene malononitriles, which react with hydroxylamine to form isoxazoles. The best results were obtained via simultaneous reaction of reagents in Gly/K_2_CO_3_ (4:1 molar) as green catalytic media at room temperature, which considered as optimal conditions. Increase in Gly/K_2_CO_3_ ratio and temperature led to a decrease in yields.

Multicomponent reaction of hydroxylamine derivatives, aldehydes and active methylene compounds is an efficient procedure to synthesize isoxazoles. Some recently proposed protocols were presented in Table 3. According to the data in the Table 3, reaction times decreased in the presence of catalysts at room temperature or under heating or UV radiation. It seems that basic catalysts are more effective than acidic equivalents. Our newly modified process provides an efficient, simple, economical, safe and eco-friendly reaction under mild conditions at acceptable products yields.

**Table 3:** Multicomponent synthesis of isoxazole derivatives

Entry	Conditions	Catalyst	Time (min)	Yield (%)	References
1	EtOH, reflux	DABCO^a^	1.5–15	65–85	[13]
2	CH_3_CN, rt	–	3600–9000	70–93	[14]
3	aq. EtOH, hν	CH_3_CO_2_Na	5–10	61–89	[15]
4	H_2_O, rt	KP^b^	30–150	85–96	[16]
5	H_2_O, rt	Boric acid	50–1440	82–95	[17]

^a^1,4-Diazabicyclo[2]octane

^b^Potassium phthalimide

The chemical structure of isoxazoles **4a**–**i** was characterized by spectral data. Nitrile groups were detected by FT-IR (~ 2220 cm^−1^) and ^13^C NMR (~ 115 ppm). Amino groups were also identified based on their absorption bands in region of ~ 3430–3330 cm^−1^ and proton chemical shifts appeared approximately 8.50 ppm.

### Biological evaluation

Based on the results obtained, isoxazoles **4a**, **b**, **d**, **e** showed broad-spectrum inhibitory activates against both Gram-positive and Gram-negative bacteria. These compounds respectively include *p*-tolyl, 4-hydroxyphenyl, 2,4-dichlorophenyl and 2,6-dichlorophenyl substituents in 3-position on isoxazole ring. Heterocycle **4b** was the only effective antibacterial agent on *Shigella flexneri*. Similarity, *Shigella dysenteriae* and *Escherichia coli* were blocked only with isoxazole **4d**. Derivatives **4c**, **f**, **g**, **h**, **i** were effective only against Gram-positive pathogens. All derivative could inhibit the growth of Gram-positive *Listeria monocytogenes*. No antifungal activity was observed with heterocyclic compounds **4c**, **e**, **f**, **g**, **h**, **i**. Isoxazoles **4b**, **d** were effective on all tested pathogenic fungi.

Free radical scavenging ability of methanolic solutions of all synthesized compounds against DPPH was determined spectrophotometrically at 517 nm. However, notable in vitro antioxidant activity was only observed in isoxazole **4i**, including pyridine-4yl substituent, with an IC_50_ = 67.51 μg ml^−1^. These effects are comparable to the effects of isoxazole derivatives with IC_50_ in the range 62.76–100.73 μg ml^−1^ [29].

## Conclusion

In summary, some novel 5-amino-isoxazole-4-carbonitriles were prepared via a green and efficient multicomponent procedure in acceptable product yields and short reaction times. Antimicrobial activity of isoxazoles was studied against a variety of bacterial and fungal pathogens. Significant inhibitory potentials were observed with compounds **4a**, **b**, **d**. Isoxazole **4i** also showed considerable antioxidant activities. These functionalized biologically active compounds could applied as prodrugs in future researches.

## Methods

### Materials

All reagents, solvents, antibiotics, DPPH and antifungal agents were purchased from commercial sources (Merck, Sigma and Aldrich), and used without further purification. The bacterial and fungal culture media were obtained from (HiMedia). Melting points were determined with Kruss type KSP1N melting point meter and are uncorrected. Reaction progress was monitored by aluminium TLC plates pre-coated by SiO_2_ with fluorescent indicator F254 using CHCl_3_/CH_3_OH (9:1, v/v) as mobile phase, which were visualized under UV radiation (254 nm). The absorption spectra were determined using a UV-2100 RAY Leigh UV–Vis spectrophotometer. FT-IR spectra of the products were collected using a Bruker Tensor-27 FT-IR spectrometer. ^1^H and ^13^C NMR spectra were recorded at 400 and 100 MHz, respectively, on a Bruker FT-NMR Ultra Shield-400 spectrometer. Elemental analyses (CHNS/O) were performed on a Thermo Finnigan Flash EA microanalyzer. DESs were prepared in various ratios of glycerol/K_2_CO_3_ according to the published procedure [30] (Additional file 1).

### General procedure for the synthesis of isoxazoles **4a**–**i**

A mixture of K_2_CO_3_ (0.140 g, 0.001 mol) and glycerol (0.360 g, 0.004 mol) was stirred at 80 °C for 2 h to form a homogenous colorless liquid. After cooling DES to room temperature, 0.001 mol each of malononitrile (**1**) (0.660 g), hydroxylamine hydrochloride (**2**) (0.070 g) and benzaldehydes **3a**–**i** (**3a**: 0.120 g, **3b**: 0.122 g, **3c**: 0.151 g, **3d**: 0.175 g, **3e**: 0.175 g, **3f**: 0.152 g; **3** **g**: 0.096 g; **3** **h**: 0.112 g; **3i**: 0.107 g) was simultaneously added to it. The reaction mixture was stirred for 20–120 min. 1 ml each of ethanol and water was added to it. The resulting precipitates were collected by filtration, washed respectively with water (5 ml) and ethanol (5 ml), and recrystallized from methanol to give pure isoxazoles **4a**–**i**.

#### 5-Amino-3-(p-tolyl)isoxazole-4-carbonitrile (**4a**)

Yield: 0.14 g, 70%; mp: 135–137 °C; reaction time: 40 min; IR (KBr) *ν*: 3408, 3337 (NH_2_), 2223 (C≡N), 1605 (C=N), 1221 (C–O–N) cm^−1^; ^1^H NMR (400 MHz, DMSO-*d*_*6*_) *δ*: 2.37 (s, 3H, CH_3_), 7.39 (d, *J* = 7.2 Hz, 2H, H-3ʹ,5ʹ), 7.82 (d, *J* = 7.2 Hz, 2H, H-2ʹ,6ʹ), 8.44 (s, 2H, NH_2_); ^13^C NMR (100 MHz, DMSO-*d*_*6*_) *δ*: 21.90 (CH_3_), 80.31 (C-4), 113.88 (C-1ʹ), 114.84 (C≡N), 129.17 (C-4ʹ), 130.58 (C-3ʹ,5ʹ), 131.12 (C-2ʹ,6ʹ), 146.12 (C-5), 161.70 (C-3); Anal. Calcd. for C_11_H_9_N_3_O: C 66.32, H 4.55, N 21.09. Found: C 66.28, H 4.52, N 21.15.

#### 5-Amino-3-(4-hydroxyphenyl)isoxazole-4-carbonitrile **(4b)**

Yield: 0.19 g, 94%; mp: 118–120 °C; reaction time: 30 min; IR (KBr) *ν*: 3509 (OH), 3426, 3335 (NH_2_), 2227 (C≡N), 1611 (C=N), 1263 (C–O–N) cm^−1^; ^1^H NMR (400 MHz, DMSO-*d*_*6*_) *δ*: 6.95 (d, *J* = 8.3 Hz, 2H, H-3ʹ,5ʹ), 7.85 (d, *J* = 7.2 Hz, 2H, H-2ʹ,6ʹ), 8.25 (s, 2H, NH_2_), 11.06 (s, 1H, OH); ^13^C NMR (100 MHz, DMSO-*d*_*6*_) *δ*: 75.53 (C-4), 114.60 (C≡N), 115.51 (C-1ʹ), 117.03 (C-3ʹ,5ʹ), 123.21 (C-5), 134.30 (C-2ʹ,6ʹ), 160.90 (C-4ʹ), 164.30 (C-3); Anal. Calcd. for C_10_H_7_N_3_O_2_: C 59.70, H 3.51, N 20.89. Found: C 59.67, H 3.58, N 20.83.

#### 5-Amino-3-(4-nitrophenyl)isoxazole-4-carbonitrile (**4c**)

Yield: 0.21 g, 92%; mp: 183–184 °C; reaction time: 35 min; IR (KBr) *ν*: 3417, 3379 (NH_2_), 2220 (C≡N), 1603 (C=N), 1541, 1361 (NO_2_), 1289 (C–O–N) cm^−1^; ^1^H NMR (400 MHz, DMSO-*d*_*6*_) *δ*: 7.92 (d, *J* = 9.4 Hz, 2H, H-2ʹ,6ʹ), 8.32 (s, 2H, NH_2_), 8.41 (m, 4H, H-3ʹ,5ʹ, NH_2_); ^13^C NMR (100 MHz, DMSO-*d*_*6*_) *δ*: 80.63 (C-4), 115.05 (C≡N), 124.23 (C-2ʹ,6ʹ), 130.97 (C-3ʹ,5ʹ), 135.98 (C-1ʹ), 146.36 (C-5), 148.00 (C-4ʹ), 152.36 (C-3); Anal. Calcd. for C_10_H_6_N_4_O_3_: C 52.18, H 2.63, N 24.34. Found: C 52.24, H 2.59, N 24.37.

#### 5-Amino-3-(2,4-dichlorophenyl)isoxazole-4-carbonitrile (**4d**)

Yield: 0.23 g, 92%; mp: 119–120 °C; reaction time: 60 min; IR (KBr) *ν*: 3426, 3347 (NH_2_), 2228 (C≡N), 1648 (C=N), 1290 (C–O–N) cm^−1^; ^1^H NMR (400 MHz, DMSO-*d*_*6*_) *δ*: 7.64 (m, 1H, H-5ʹ), 7.86 (s, 1H, H-3ʹ), 8.01 (d, *J* = 7.9 Hz, 1H, H-6ʹ), 8.58 (s, 2H, NH_2_); ^13^C NMR (100 MHz, DMSO-*d*_*6*_) *δ*: 87.50 (C-4), 113.76 (C≡N), 128.28 (C-5ʹ), 128.75 (C-1ʹ), 129.69 (C-6ʹ), 130.47 (C-3ʹ), 131.38 (C-2ʹ) 139.18 (C-4ʹ), 144.13 (C-5), 157.13 (C-3); Anal. Calcd. for C_10_H_6_N_4_O_3_: C 52.18, H 2.63, N 24.34. Found: C 52.24, H 2.59, N 24.37.

#### 5-Amino-3-(2,6-dichlorophenyl)isoxazole-4-carbonitrile (**4e**)

Yield: 0.22 g, 88%; mp: 150–152 °C; reaction time: 50 min; IR (KBr) *ν*: 3432, 3358 (NH_2_), 2221 (C≡N), 1647 (C=N), 1299 (C–O–N) cm^−1^; ^1^H NMR (400 MHz, DMSO-*d*_*6*_) *δ*: 7.38 (d, *J* = 7.1 Hz, 1H, H-4ʹ), 7.48 (d, *J* = 7.1 Hz, 2H, H-3ʹ,5ʹ), 8.18 (s, 2H, NH_2_); ^13^C NMR (100 MHz, DMSO-*d*_*6*_) *δ*: 82.57 (C-4), 113.10 (C≡N), 129.31 (C-3ʹ,5ʹ), 129.78 (C-1ʹ), 131.37 (C-2ʹ,6ʹ), 134.32 (C-4ʹ), 144.20 (C-5), 155.25 (C-3); Anal. Calcd. for C_10_H_6_N_4_O_3_: C 52.18, H 2.63, N 24.34. Found: C 52.20, H 2.66, N 24.29.

#### 5-Amino-3-(2-hydroxy-3-methoxyphenyl)isoxazole-4-carbonitrile (**4f**)

Yield: 0.17 g, 75%; mp: 220–222 °C; reaction time: 120 min; IR (KBr) *ν*: 3509 (OH), 3408, 3341 (NH_2_), 2230 (C≡N), 1606 (C=N), 1287 (C–O–N) cm^−1^; ^1^H NMR (400 MHz, DMSO-*d*_*6*_) *δ*: 3.87 (s, 3H, CH_3_), 7.27–7.39 (m, 3H, H-4ʹ,5ʹ,6ʹ), 8.38 (s, 2H, NH_2_), 10.31 (s, 1H, OH); ^13^C NMR (100 MHz, DMSO-*d*_*6*_) *δ*: 56.67 (CH_3_), 102.74 (C-4), 114.97 (C≡N), 117.82 (C-4ʹ), 118.37 (C-1ʹ), 121.16 (C-5ʹ), 125.82 (C-6ʹ), 143.68 (C-2ʹ), 146.87 (C-5), 154.08 (C-3ʹ), 157.00 (C-3); Anal. Calcd. for C_11_H_9_N_3_O_3_: C 57.14, H 3.92, N 18.17. Found: C 57.19, H 3.94, N 18.13.

#### 5-Amino-3-(furan-2-yl)isoxazole-4-carbonitrile (**4g**)

Yield: 0.13 g, 85%; mp: 270–272 °C (dec.); reaction time: 25 min; IR (KBr) *ν*: 3425, 3369 (NH_2_), 2221 (C≡N), 1601 (C=N), 1289 (C–O–N) cm^−1^; ^1^H NMR (400 MHz, DMSO-*d*_*6*_) *δ*: 6.77 (m, 1H, H-3ʹ), 7.23 (m, 1H, H-2ʹ), 8.02 (m, 1H, H-4ʹ), 8.30 (s, 2H, NH_2_); ^13^C NMR (100 MHz, DMSO-*d*_*6*_) *δ*: 76.90 (C-4), 109.35 (C-2ʹ), 113.05 (C-3ʹ), 115.52 (C≡N), 135.12 (C-4ʹ), 146.31 (C-3), 153.00 (C-1ʹ), 160.29 (C-5); Anal. Calcd. for C_8_H_5_N_3_O_2_: C 54.86, H 2.88, N 23.99. Found: C 54.81, H 2.90, N 24.03.

#### 5-Amino-3-(thiophen-2-yl)isoxazole-4-carbonitrile (**4h**)

Yield: 0.15 g, 79%; mp: 249–251 °C (dec.) (Lit. [31]: 225–226 °C); reaction time: 60 min; IR (KBr) *ν*: 3425, 3363 (NH_2_), 2204 (C≡N), 1600 (C=N), 1281 (C–O–N) cm^−1^; ^1^H NMR (400 MHz, DMSO-*d*_*6*_) *δ*: 7.25 (m, 1H, H-3ʹ), 7.45 (m, 1H, H-2ʹ), 7.87 (m, 1H, H-4ʹ), 8.34 (s, 2H, NH_2_); ^13^C NMR (100 MHz, DMSO-*d*_*6*_) *δ*: 80.52 (C-4), 115.26 (C≡N), 128.16 (C-2ʹ), 130.63 (C-3ʹ), 131.21 (C-4ʹ), 141.09 (C-1ʹ), 152.56 (C-3), 161.60 (C-5); Anal. Calcd. for C_8_H_5_N_3_OS: C 50.25, H 2.64, N 21.98, S 16.77. Found: C 50.31, H 2.61, N 22.01, S 16.71.

#### 5-Amino-3-(pyridin-4-yl)isoxazole-4-carbonitrile (**4i**)

Yield: 0.17 g, 91%; mp: 255–257 °C (dec.); reaction time: 20 min; IR (KBr) *ν*: 3434, 3356 (NH_2_), 2216 (C≡N), 1602 (C=N), 1288 (C–O–N) cm^−1^; ^1^H NMR (400 MHz, DMSO-*d*_*6*_) *δ*: 7.37–7.55 (m, 2H, H-2ʹ,6ʹ), 8.45 (s, 2H, NH_2_), 8.76 (d, *J* = 7.5 Hz, 2H, H-3ʹ,5ʹ); ^13^C NMR (100 MHz, DMSO-*d*_*6*_) *δ*: 80.03 (C-4), 114.82 (C≡N), 123.80 (C-2ʹ,6ʹ), 142.69 (C-1ʹ), 150.39 (C-3ʹ,5ʹ), 152.43 (C-3), 161.23 (C-5); Anal. Calcd. for C_9_H_6_N_4_O: C 58.06, H 3.25, N 30.09. Found: C 58.01, H 3.27, N 30.15.

### Biological assay

Gram-negative bacterial strains including *Pseudomonas aeruginosa* (PTCC 1310), *Shigella flexneri* (PTCC 1234), *Shigella dysenteriae* (PTCC 1188), *Klebsiella pneumoniae* (PTCC 1290), *Acinetobacter baumannii* (PTCC 1855), *Escherichia coli* (PTCC 1399), Gram-positive bacterial strains including *Streptococcus pyogenes* (PTCC 1447), *Streptococcus agalactiae* (PTCC 1768), *Streptococcus pneumoniae* (PTCC 1240), *Staphylococcus epidermidis* (PTCC 1435), *Rhodococcus equi* (PTCC 1633), *Listeria monocytogenes* (PTCC 1297), *Streptococcus equinus* (PTCC 1445), *Bacillus subtilis* subsp. *spizizenii* (PTCC 1023), *Bacillus thuringiensis* subsp. *kurstaki* (PTCC 1494), *Staphylococcus aureus* (PTCC 1189), *Bacillus cereus* (PTCC 1665) and fungi including *Aspergillus fumigatus* (PTCC 5009), *Candida albicans* (PTCC 5027) and *Fusarium oxysporum* (PTCC 5115) were prepared from the Persian Type Culture Collection (PTCC), Karaj, Iran. All biological tests were repeated at least three times. The results were reported as the mean of three independent experiments.

### MIC determination

Broth microdilution methods according to CLSI guidelines M07-A9 and M27-A2 were used for the determination of MIC values [32, 33]. Bacterial and fungal suspensions at 0.5 McFarland standard were prepared in MHB and SDB, respectively. They were diluted to 150 and 250 times with MHB and SDB, respectively. 20 μl of each isoxazoles **4a**–**i** with concentration of 20,480 μg ml^−1^ in DMSO was added to first and second wells in a row of a 96-well microtiter plate. 20 μl DMSO was added to wells 2–12, and two-fold serial dilutions were carried out in them. 170 μl of MHA or SDB with 10 μl of diluted microbial suspensions were added to all wells. Finally, a concentration range of 2048–1 μg ml^−1^ of the derivatives was prepared in each row; in addition, the concentration of DMSO did not exceed 10% (v/v). Microtiter plates were incubated with shaking at 100 rpm at 37 °C for 24 h. Fungi must be incubated in the relative humidity (45–55%). The lowest concentration of derivatives that resulted in no visible turbidity was considered as the MIC value.

### MBC and MFC determination

Time-kill test according to CLSI guideline M26-A was applied to determine MBC and MFC values [32, 33]. Samples of all wells that showed no growth in the MIC test, were cultured in MHA or SDA media plates. Dishes were incubated at 37 °C for another 24 h under similar conditions. The MBC or MFC was identified as the lowest concentration of derivatives at which no microorganisms survived.

### IC_50_ identification

Free radical scavenging activity of all synthesized heterocycles were evaluated against DPPH [34]. 1 ml of various concentrations of all compounds (25, 50, 75, and 100 µg ml^−1^) in methanol was added to 4 ml of 0.004% (w/v) methanolic solution of freshly prepared DPPH. Solutions were shaken and left to stand for 30 min at room temperature in darkness. A solution including 1 ml of methanol and 4 ml of 0.004% (w/v) methanolic solution of DPPH was considered as blank sample. The absorbance was read at 517 nm against methanol. It should be noted that the concentration of solute is decreased to one-fifth after a dilution. The inhibition percentage (I%) for scavenging DPPH free radical was calculated according to the following equation:

$${\text{I}}\% \, = \,\left[ {{{\left( {{\text{A}}\;{\text{blank}} - {\text{A}}\;{\text{sample}}} \right)} \mathord{\left/ {\vphantom {{\left( {{\text{A}}\;{\text{blank}} - {\text{A}}\;{\text{sample}}} \right)} {\left( {{\text{A}}\;{\text{blank}}} \right)}}} \right. \kern-0pt} {\left( {{\text{A}}\;{\text{blank}}} \right)}}} \right]\, \times \,100.$$

where “A blank” and “A sample” are the absorbance of control and sample solutions, respectively. A graph of inhibition percentage vs concentration (where X axis is concentration and Y axis is I%). Equation of straight lines was determined. The half maximal inhibitory concentration (IC_50_) is “x” in equation y = mx + b while y = 50.

## Additional file

**Additional file 1.** The copies of ^1^H NMR and ^13^C NMR spectra for isoxazoles **4a–i**.

## Abbreviations

MHB: Mueller–Hinton broth
SDB: sabouraud dextrose broth
MHA: Mueller–Hinton agar
SDA: sabouraud dextrose agar
DPPH: 1,1-diphenylpicrylhydrazyl
HIV: the human immunodeficiency virus
DES: deep eutectic solvent
MIC: the minimum inhibitory concentration
MBC: the minimum bactericidal concentration
MFC: the minimum fungicidal concentration
FT-IR: Fourier Transform infrared
^1^H NMR: proton nuclear magnetic resonance
^13^C NMR: carbon-13 nuclear magnetic resonance
UV: ultraviolet
IC_50_: the half maximal inhibitory concentration
PTCC: Persian Type Culture Collection
CLSI: Clinical and Laboratory Standards Institute
