# Supplementary material for: Green multicomponent synthesis, antimicrobial and antioxidant evaluation of novel 5-amino-isoxazole-4-carbonitriles
Source: Chem Cent J. 2018 Nov 15;12:114. doi: 10.1186/s13065-018-0488-0 (PMC6768021; doi:10.1186/s13065-018-0488-0)

Additional file 1

**Green multicomponent synthesis, antimicrobial and antioxidant evaluation of novel 5-amino-isoxazole-4-carbonitriles**

Hamid Beyzaei^1*^, Mahboubeh Kamali Deljoo^1^, Reza Aryan^1^, Behzad Ghasemi^2^, Mohammad Mehdi Zahedi^3^ and Mohammadreza Moghaddam-Manesh^4^

^1^ *Department of Chemistry, Faculty of Science, University of Zabol, Zabol, Iran*

^2^ *Torbat Jam Faculty of Medical Sciences, Torbat Jam, Iran*

^3^ *Department of Chemistry, University of Saskatchewan, 110 Science Place, Saskatoon, SK S7N 5C9, Canada*

^4^ *Young Researchers and Elite Club, Kerman Branch, Islamic Azad University, Kerman, Iran*

*E-mail address*: hbeyzaei@yahoo.com (Mahboubeh Kamali Deljoo).

*E-mail address*: rezaaryanchemist@yahoo.com (Reza Aryan).

*E-mail address*: behzad.ghasemi99@gmail.com [(](mailto:cjdxnxyzx@sina.com (Qinglai)Behzad Ghasemi).

*E-mail address*: mmehdi_zahedi@yahoo.com (Mohammad Mehdi Zahedi).

*E-mail address*: mrm.manesh@gmail.com (Mohammadreza Moghaddam-Manesh).

^*^Corresponding author. Tel/Fax: +98 543-1232180.

*E-mail address*: hbeyzaei@yahoo.com (Hamid Beyzaei).

**^1^H NMR and ^13^C NMR spectra**

**1. Spectra of compound 4a**

**^1^H-NMR Spectrum**


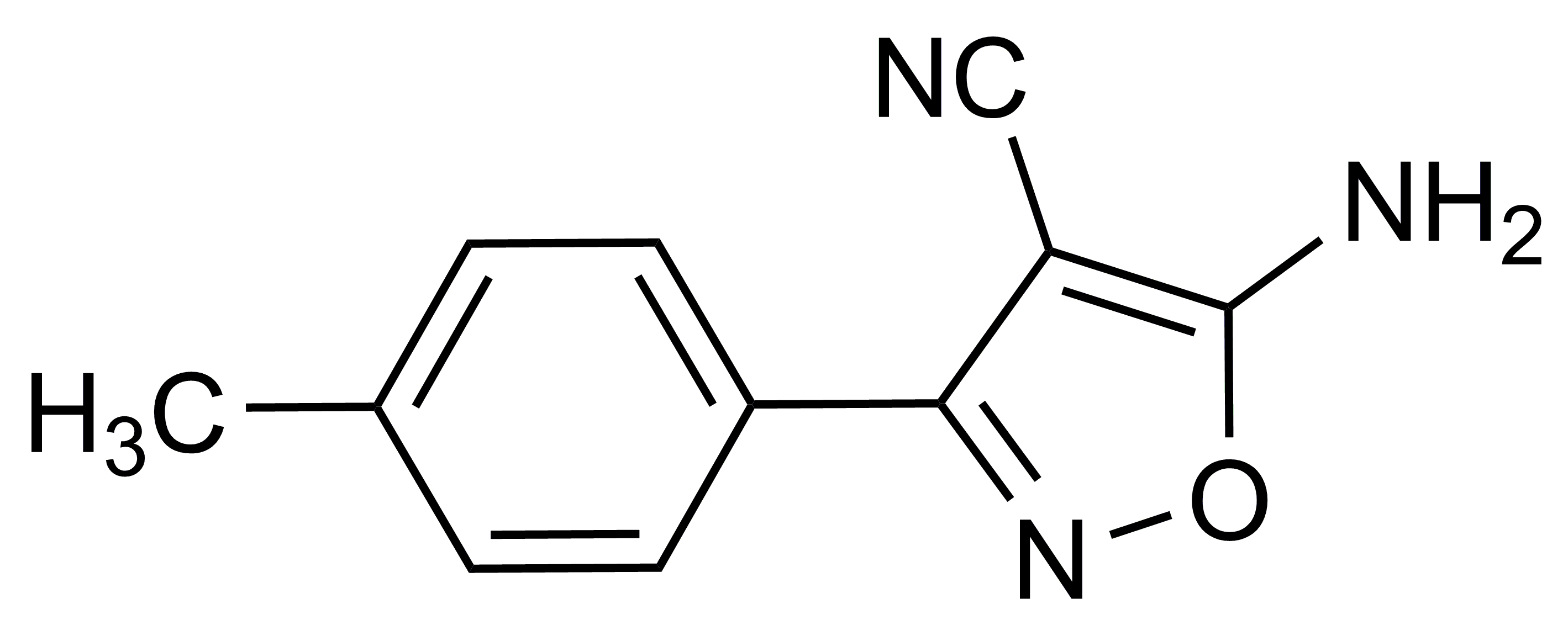

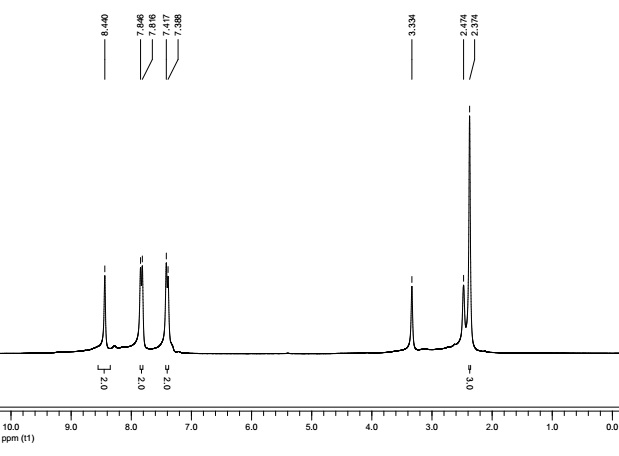


**^13^C NMR Spectrum**


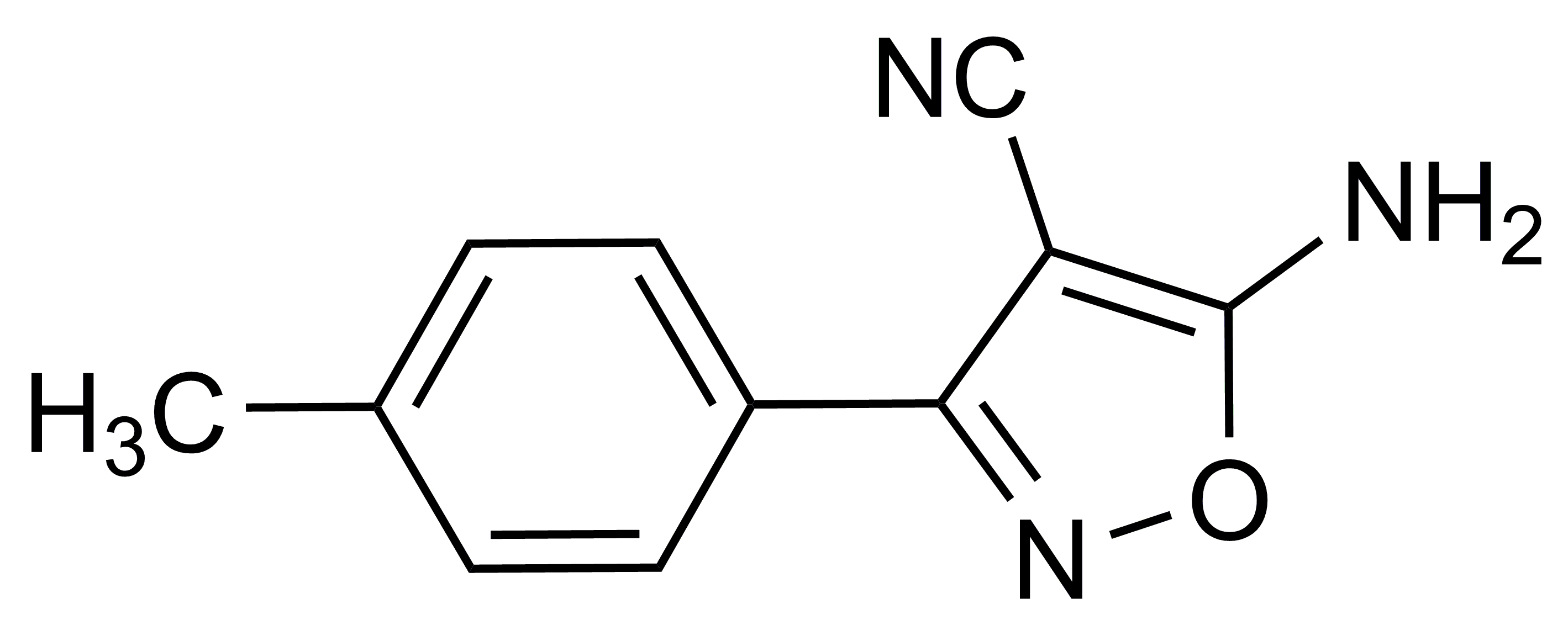

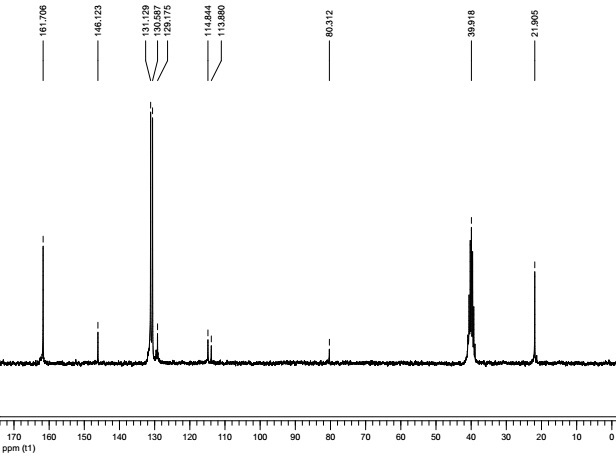


**2. Spectra of compound 4b**

**^1^H-NMR Spectrum**

**
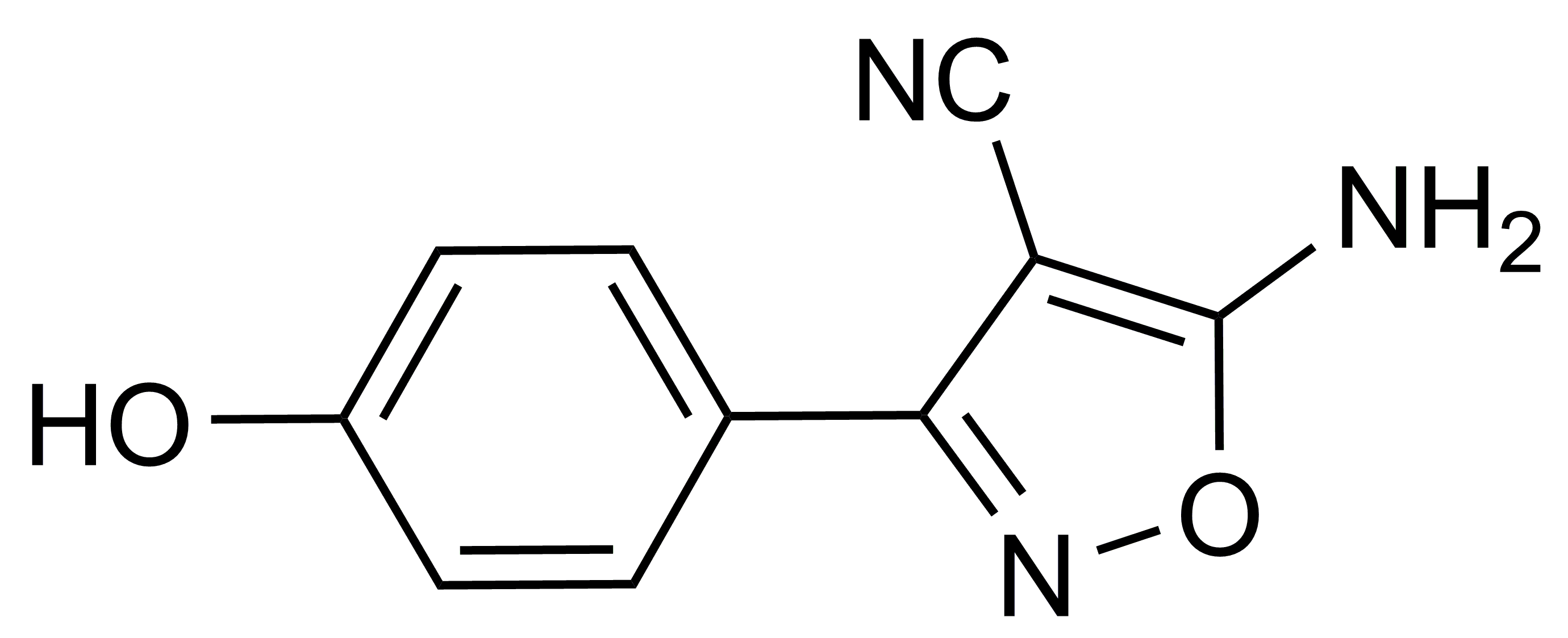

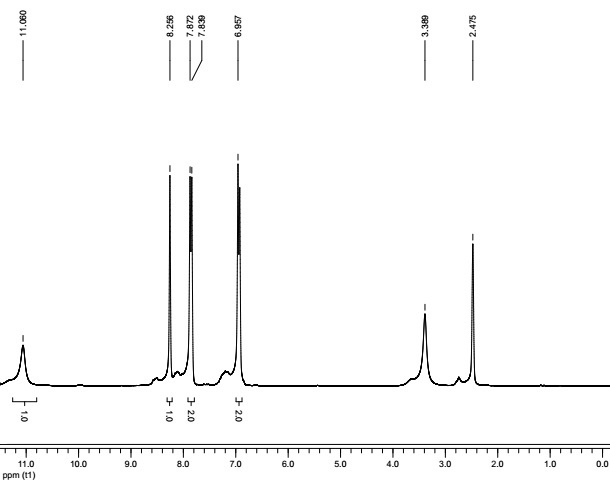
**

**^13^C NMR Spectrum**

**
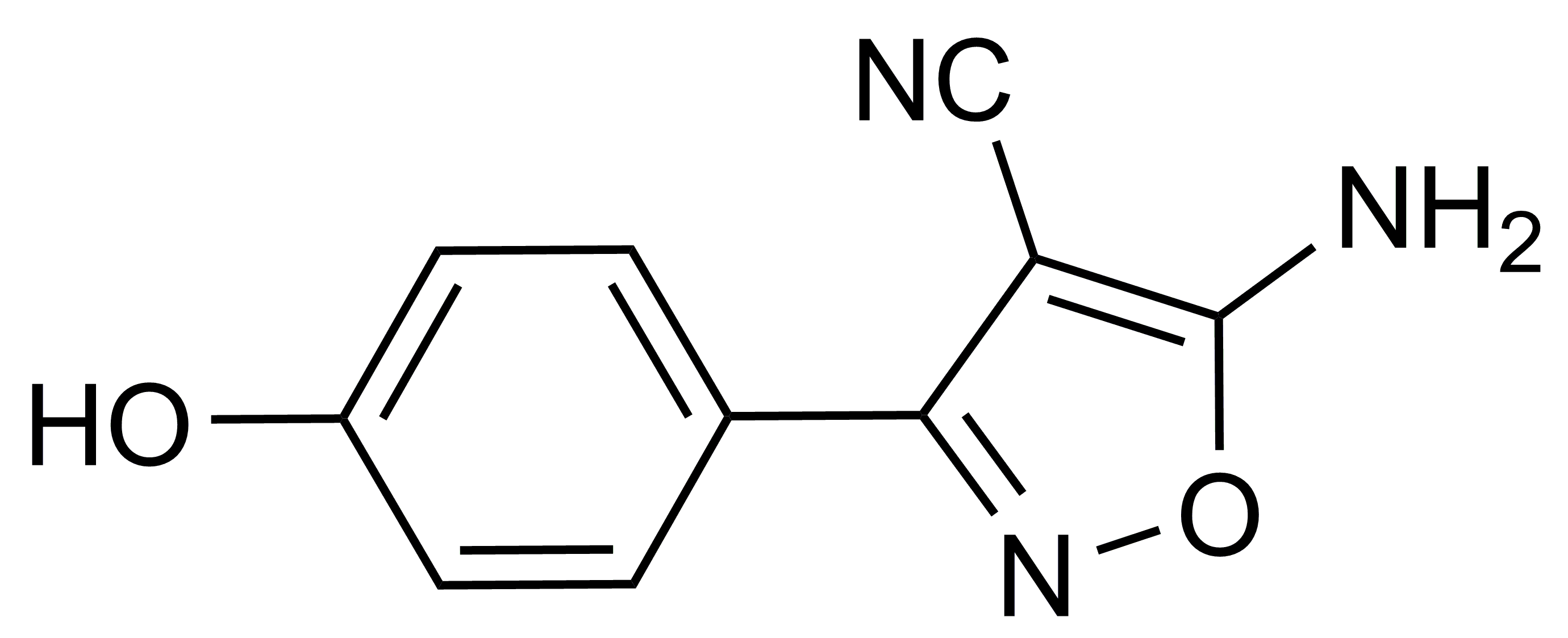

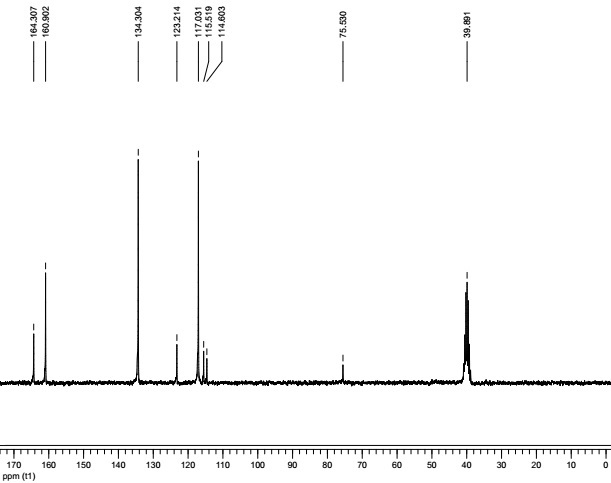
**

**3. Spectra of compound 4c**

**^1^H-NMR Spectrum**


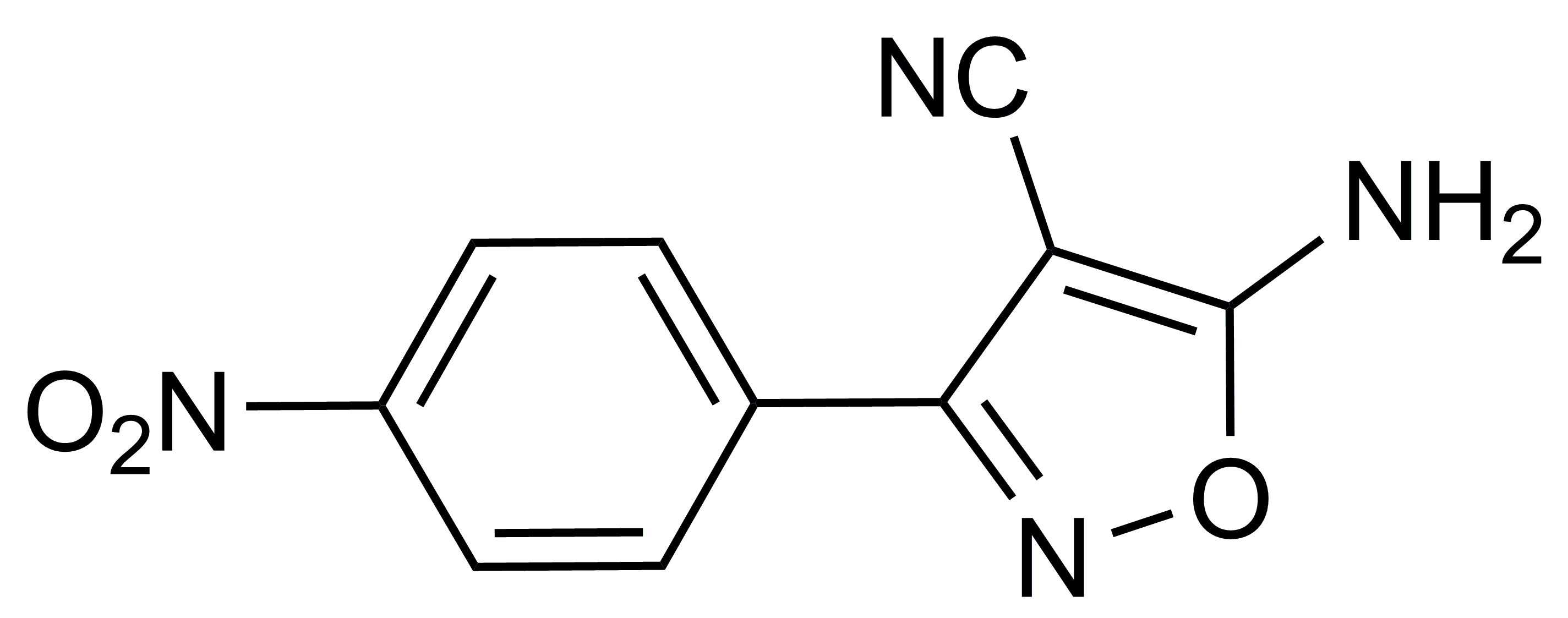

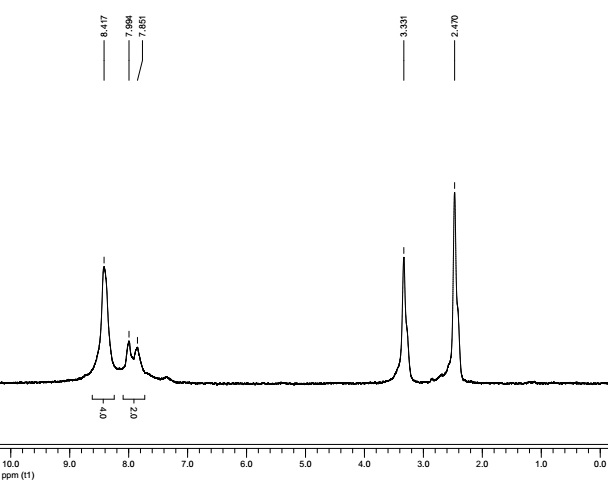


**^13^C NMR Spectrum**


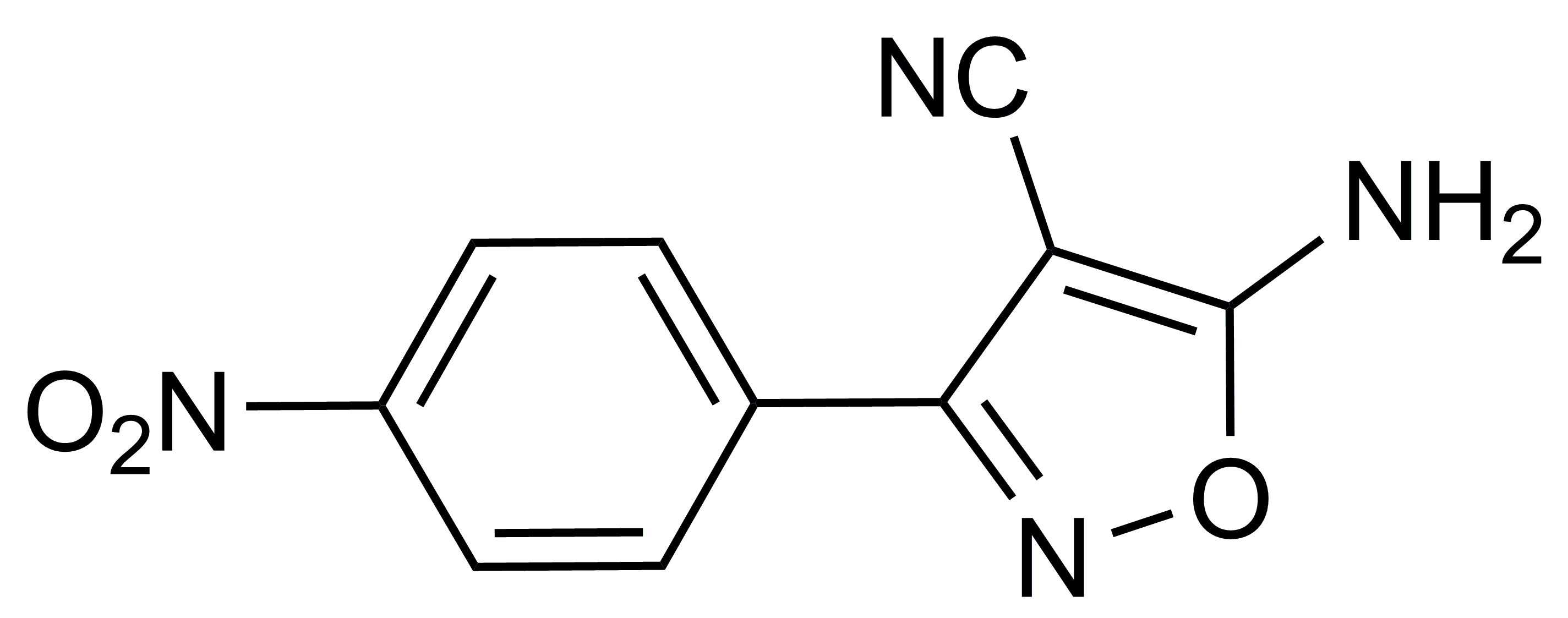

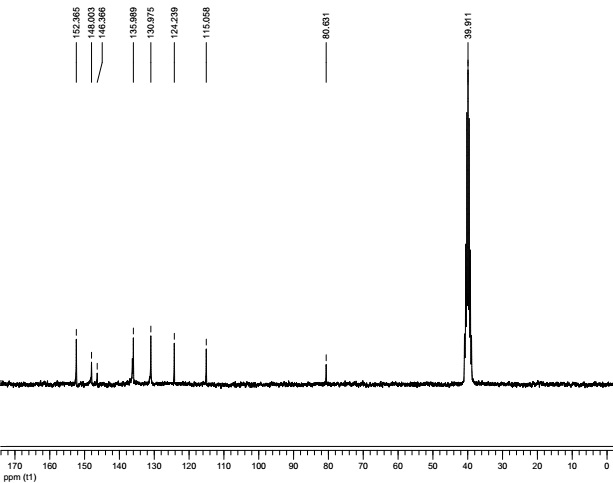


**4. Spectra of compound 4d**

**^1^H-NMR Spectrum**

**
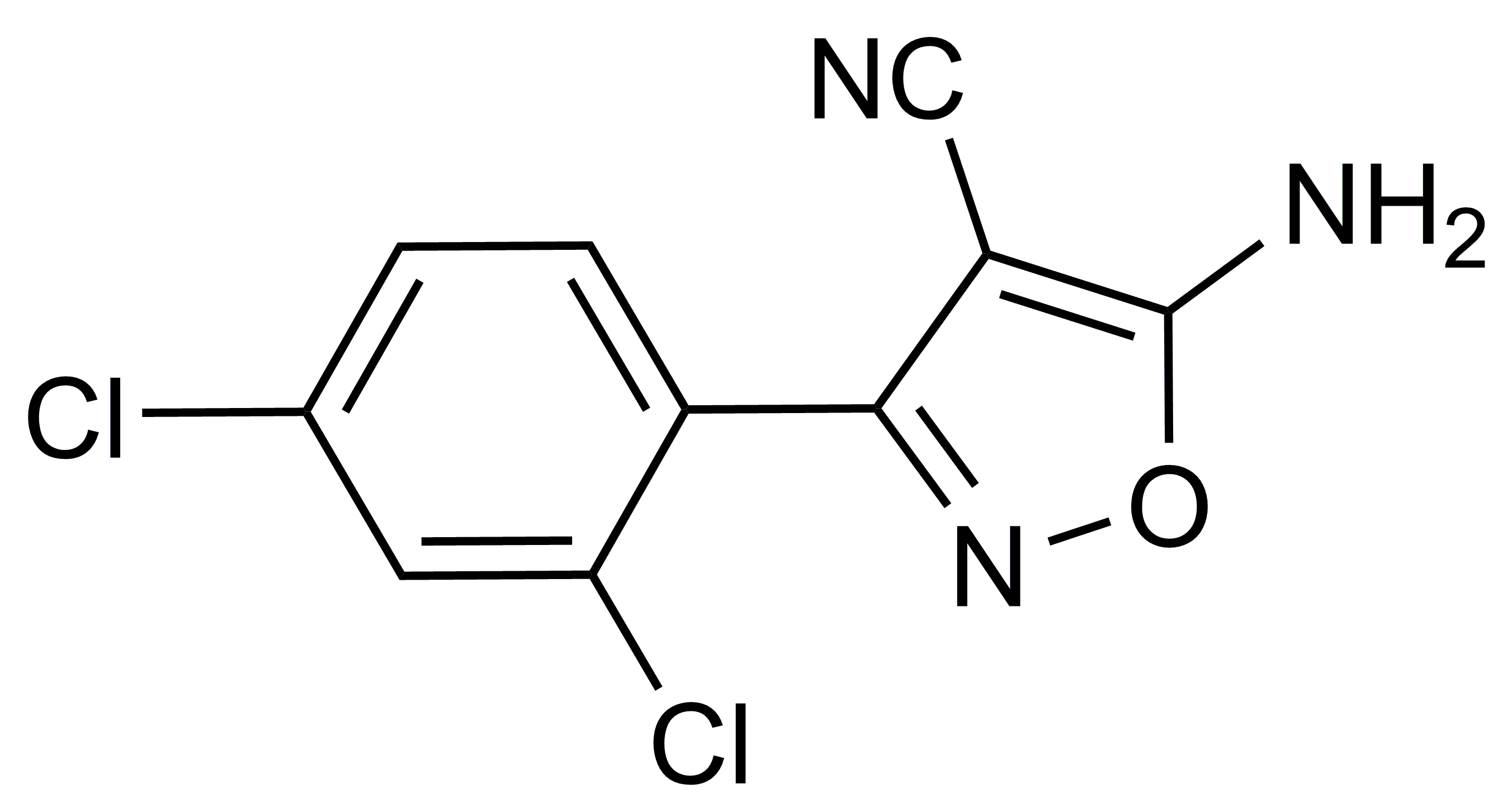

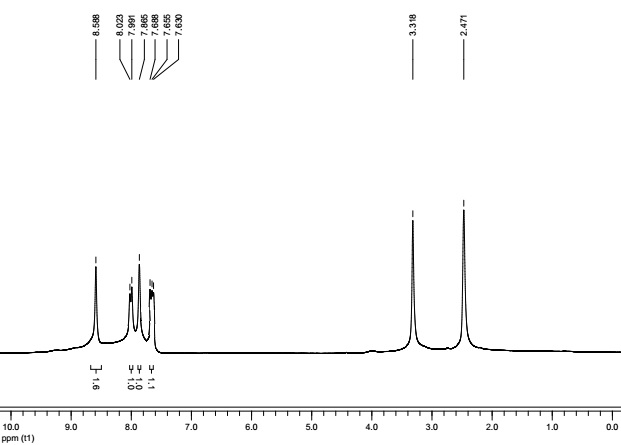
**

**^13^C NMR Spectrum**


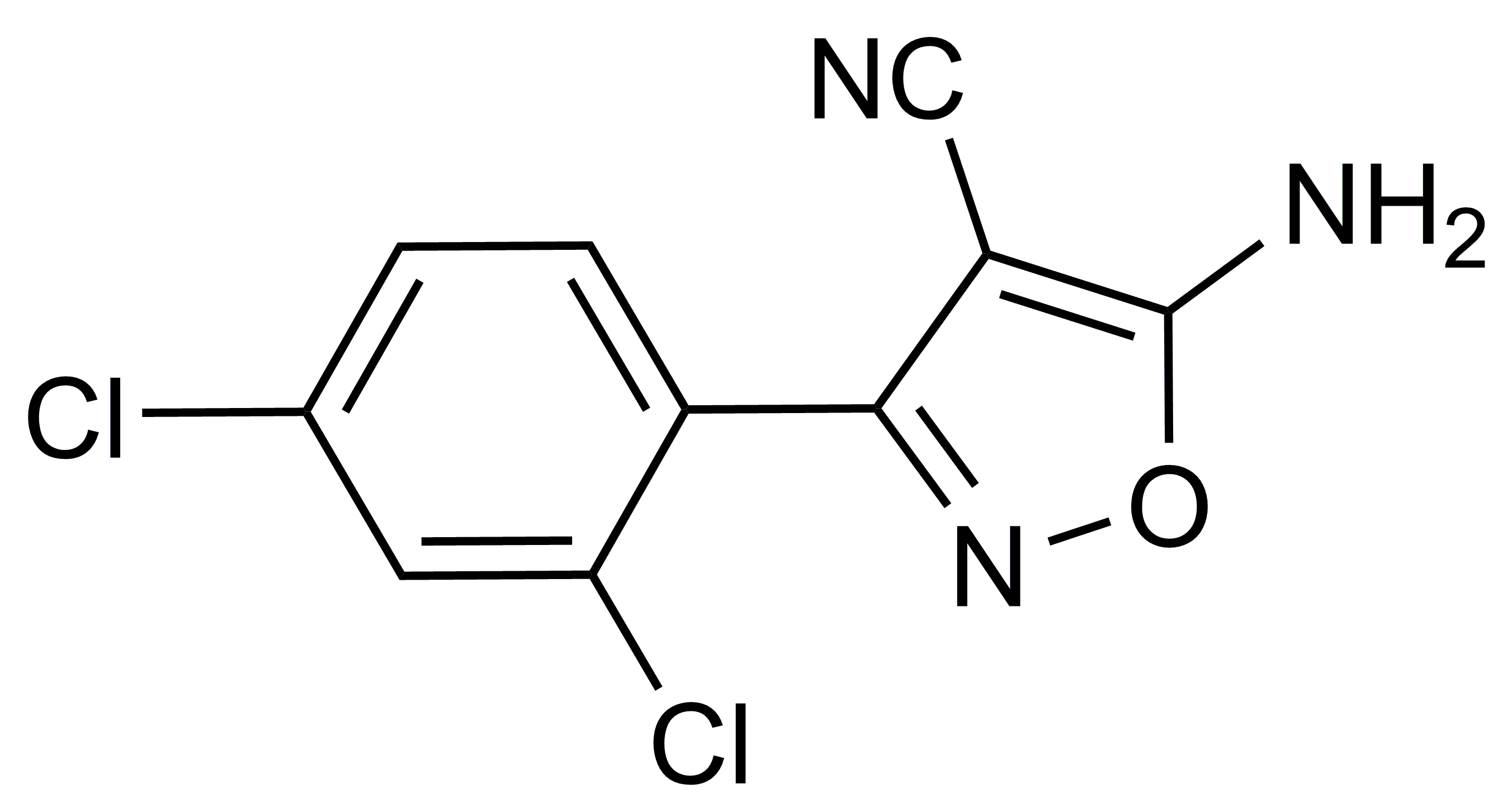

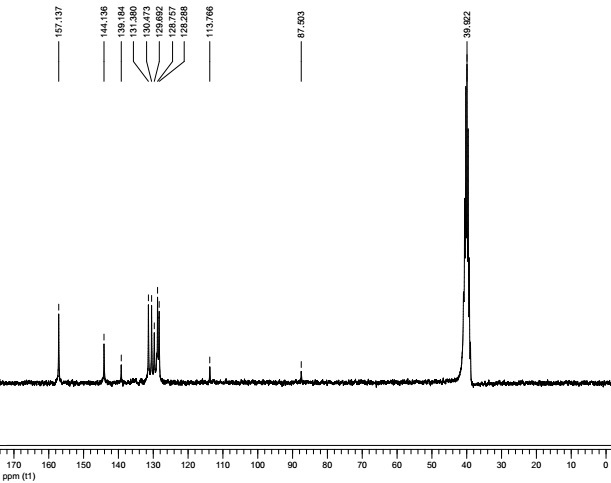


**5. Spectra of compound 4e**

**^1^H-NMR Spectrum**


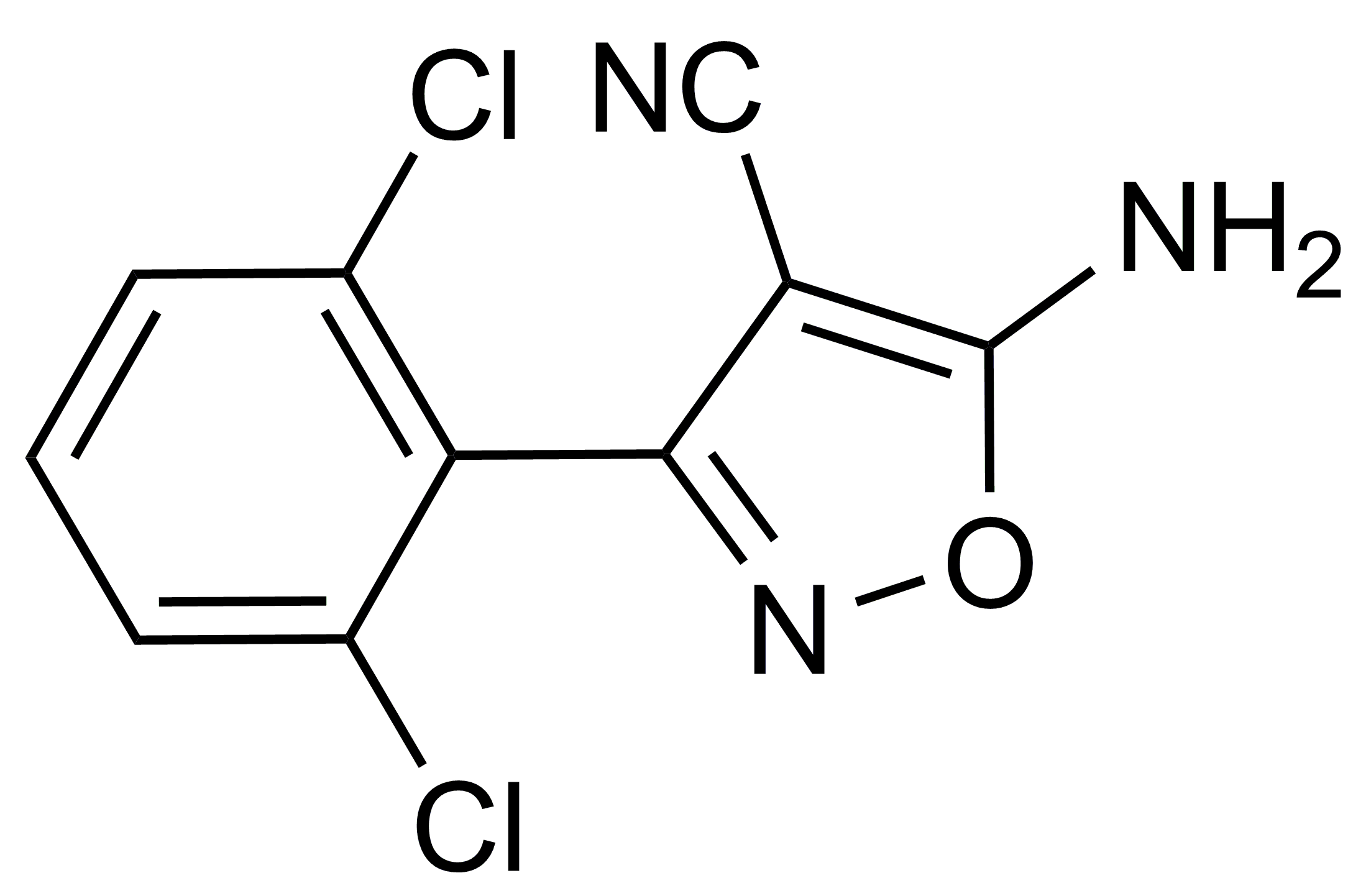

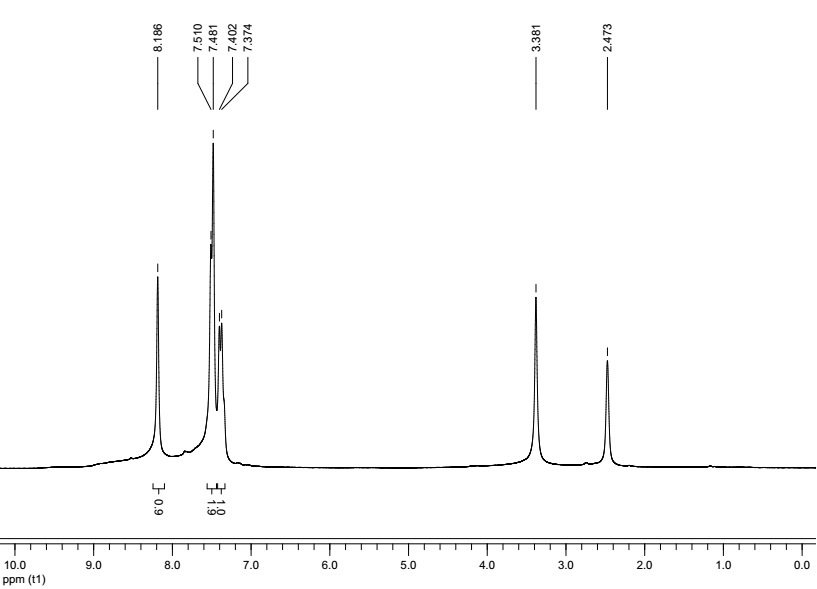


**^13^C NMR Spectrum**


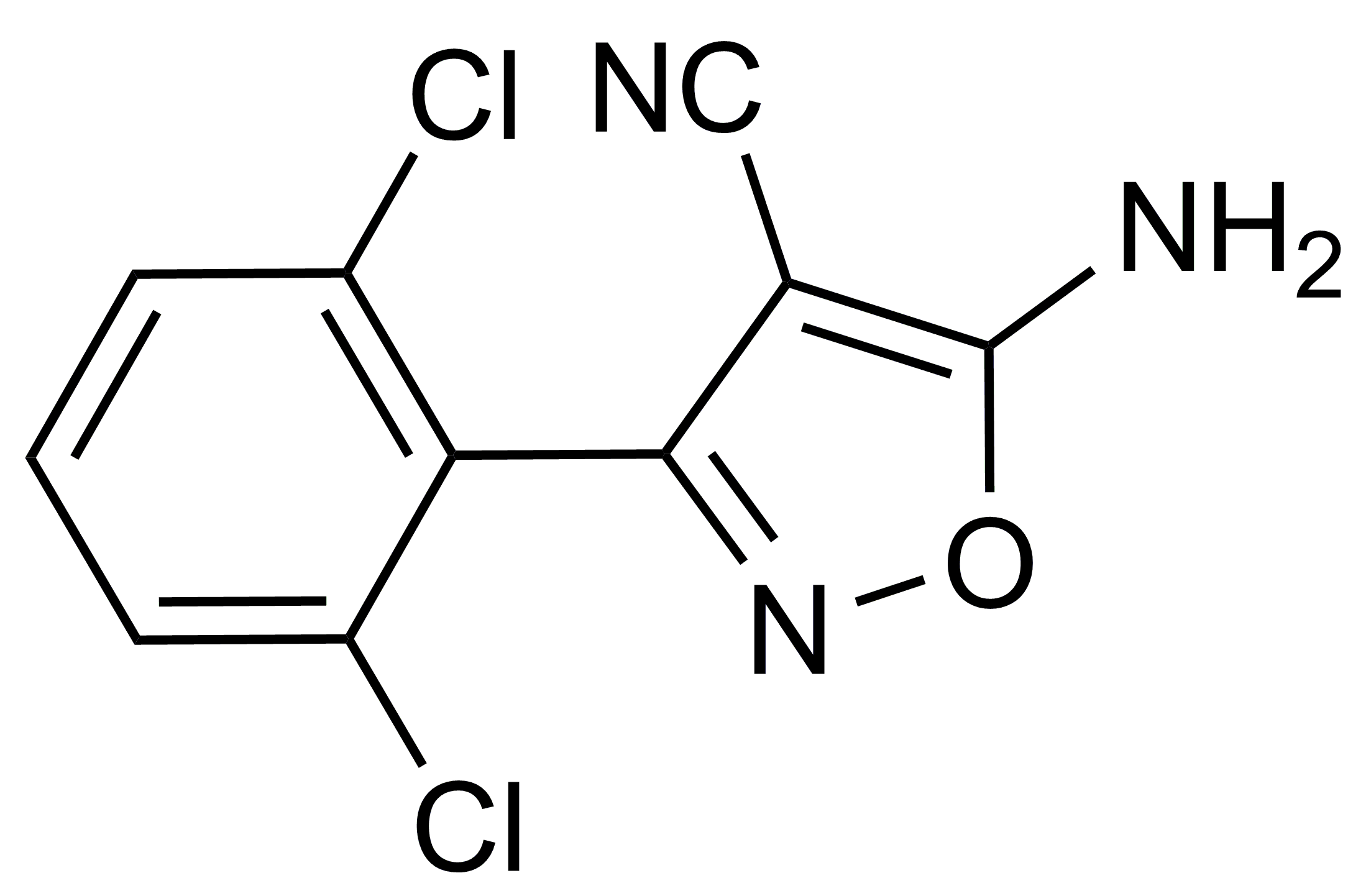

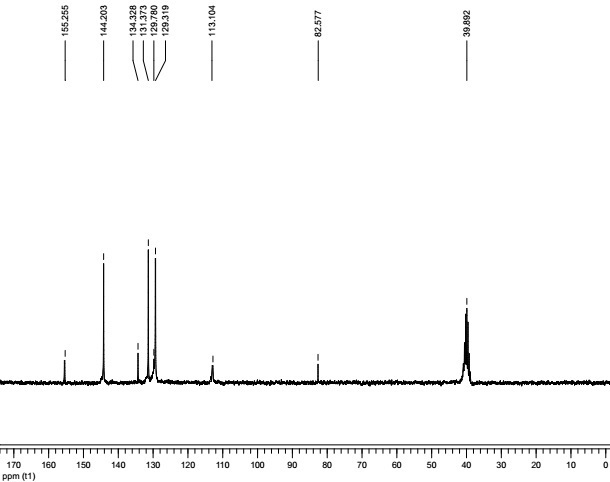


**6. Spectra of compound 4f**

**^1^H-NMR Spectrum**


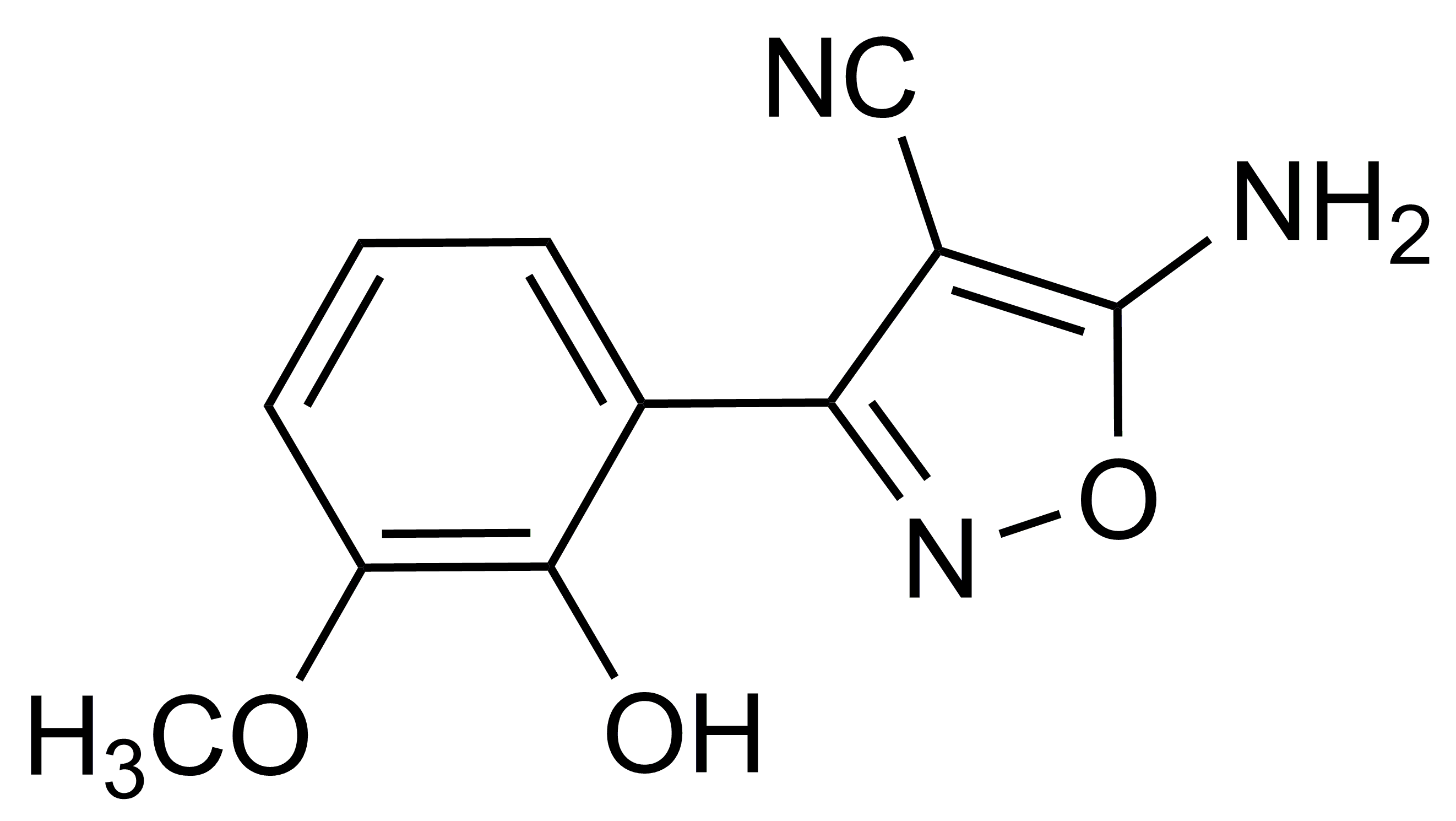

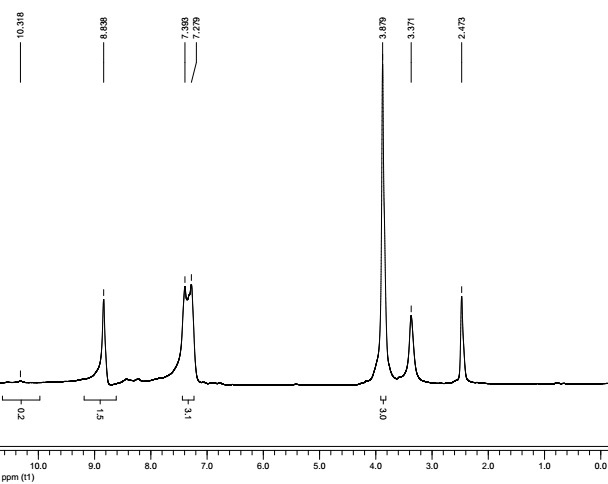


**^13^C NMR Spectrum**


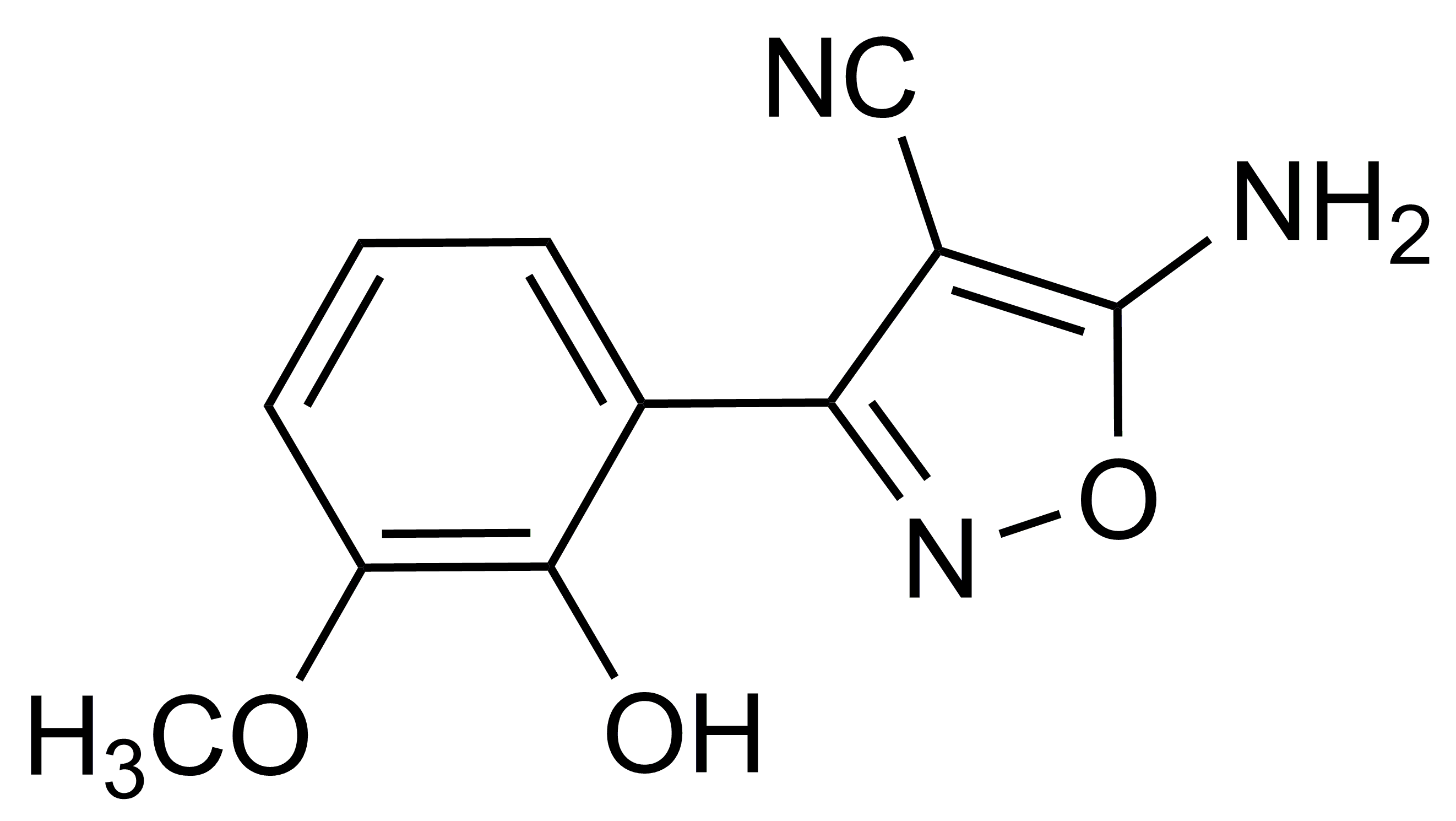

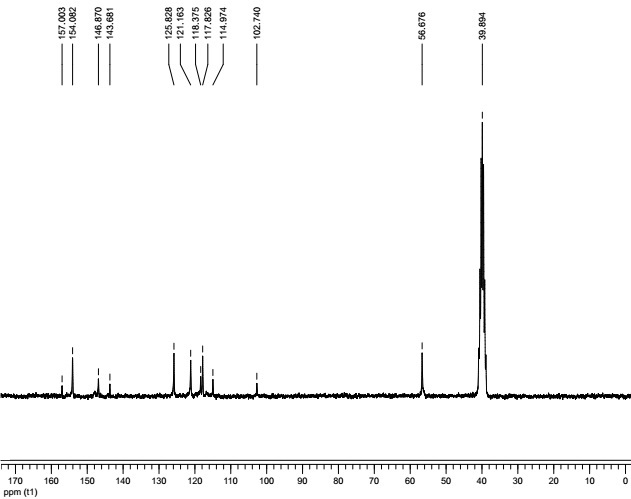


**7. Spectra of compound 4g**

**^1^H-NMR Spectrum**

**
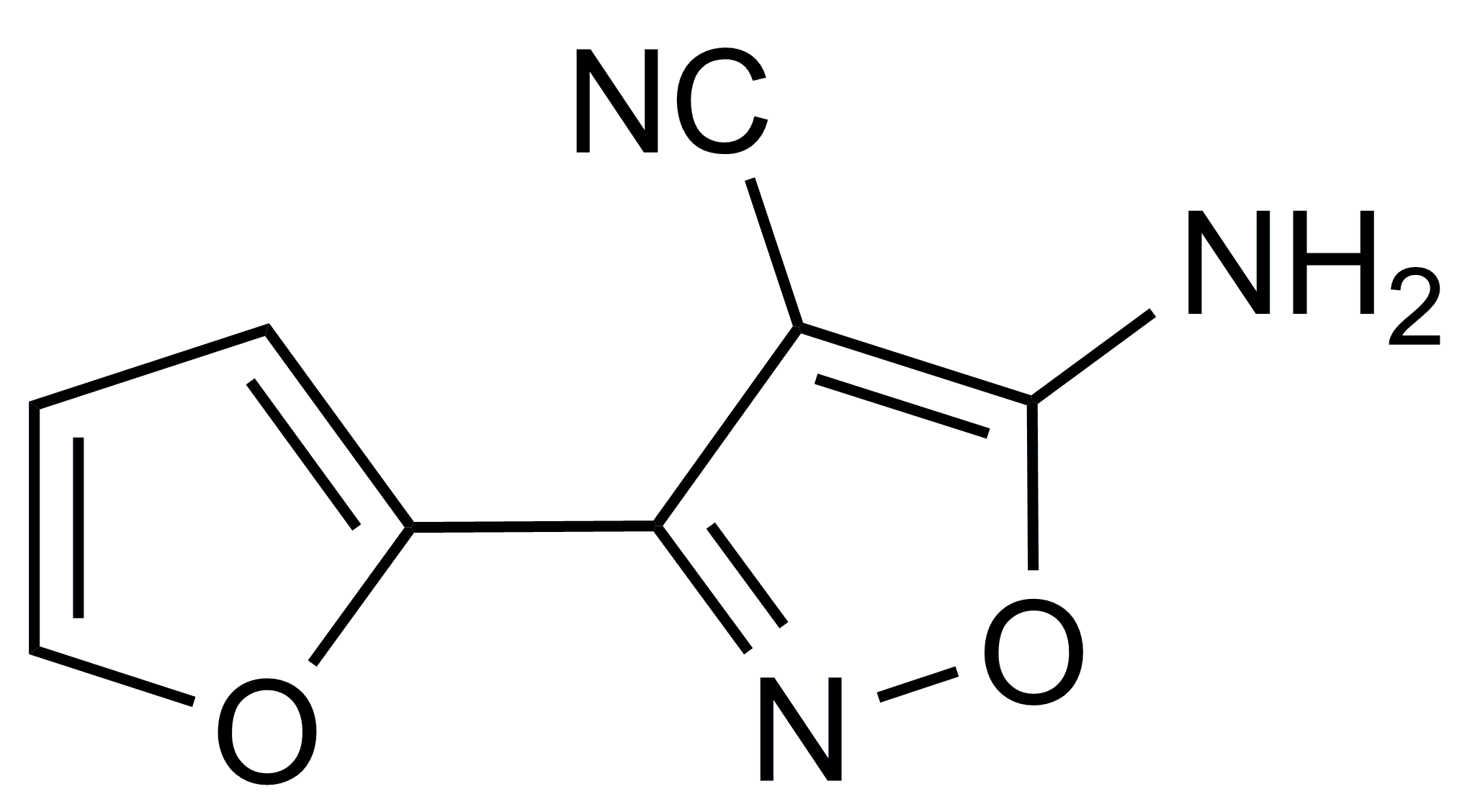

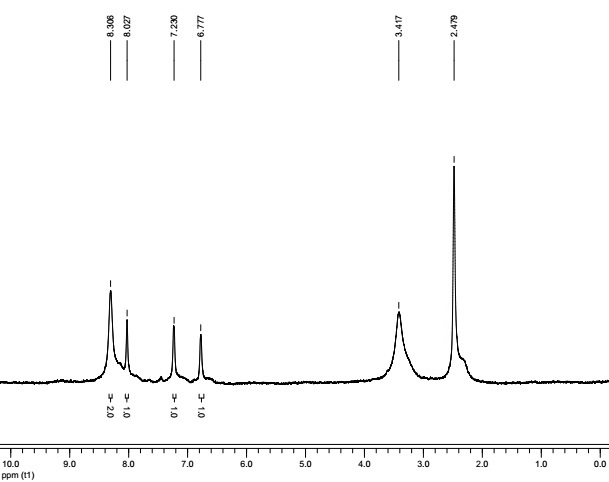
**

**^13^C NMR Spectrum**


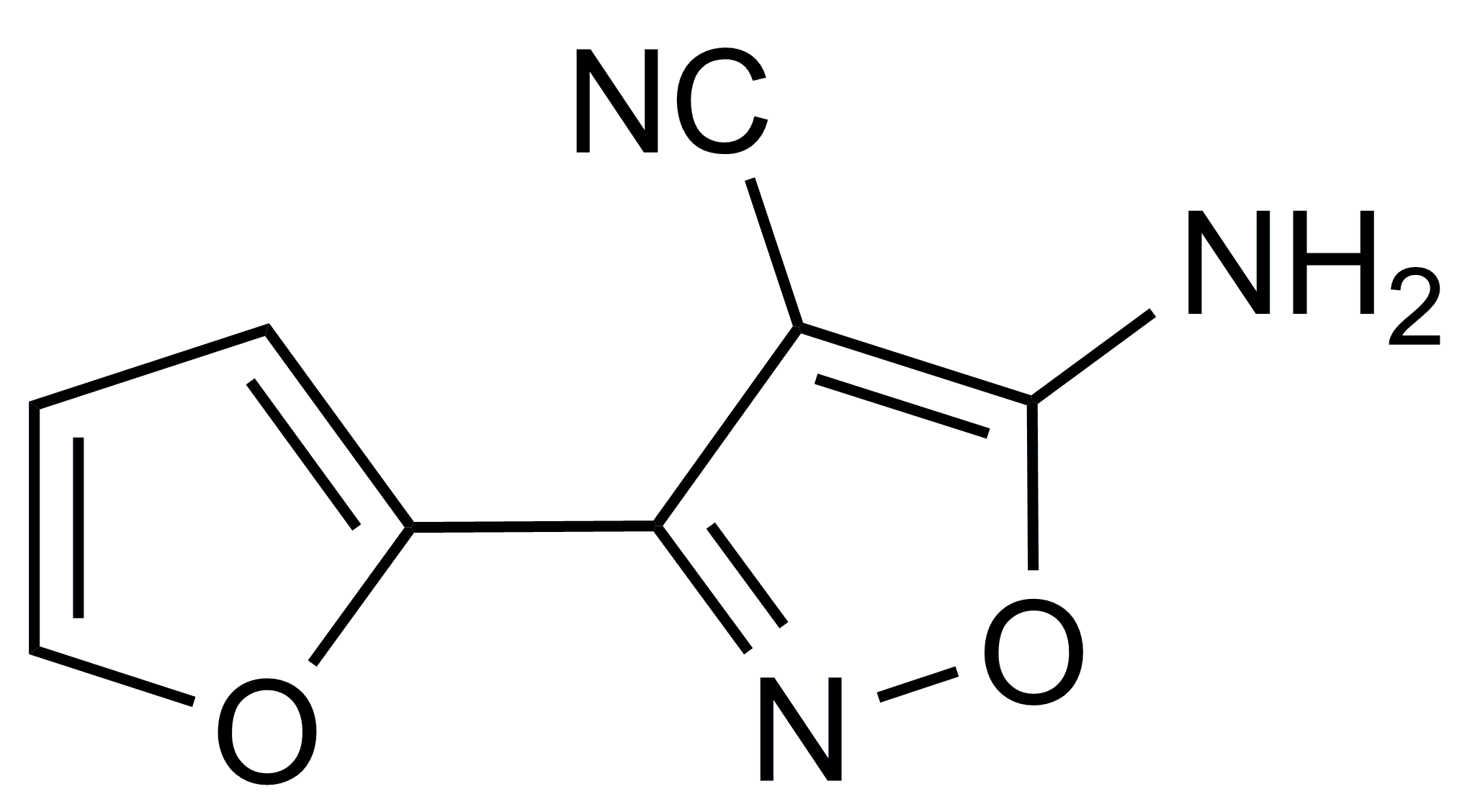

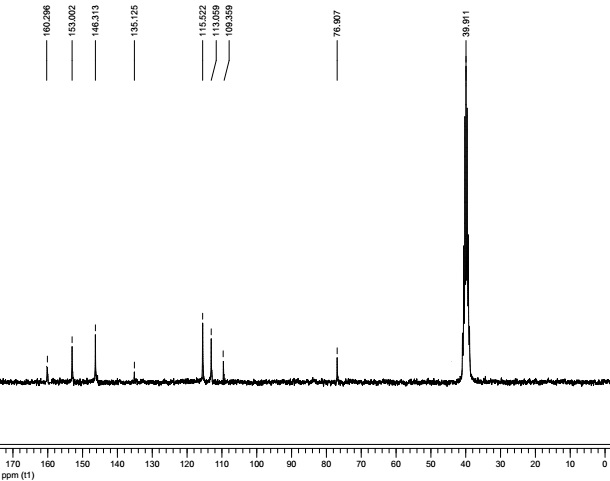


**8. Spectra of compound 4h**

**^1^H-NMR Spectrum**

**
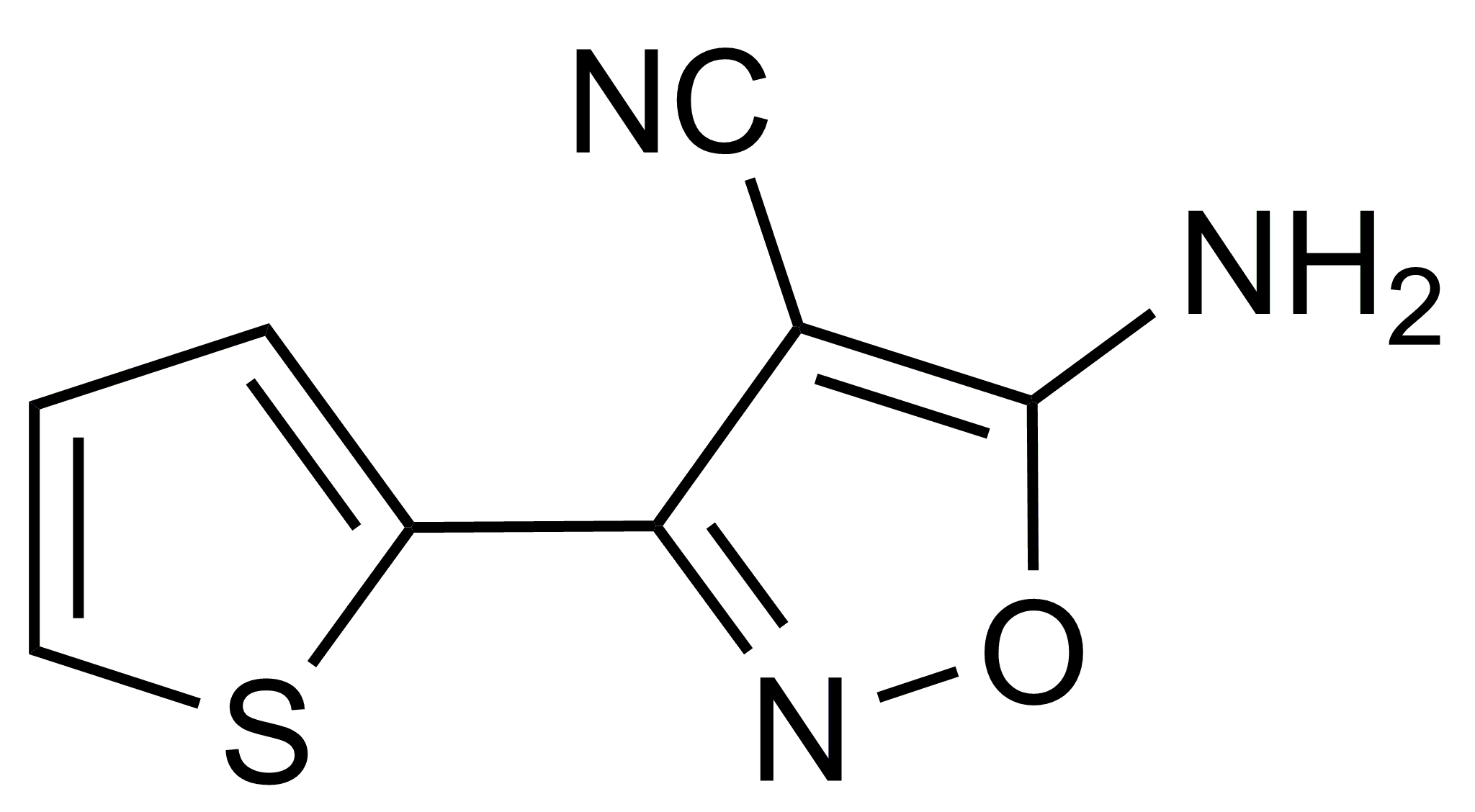

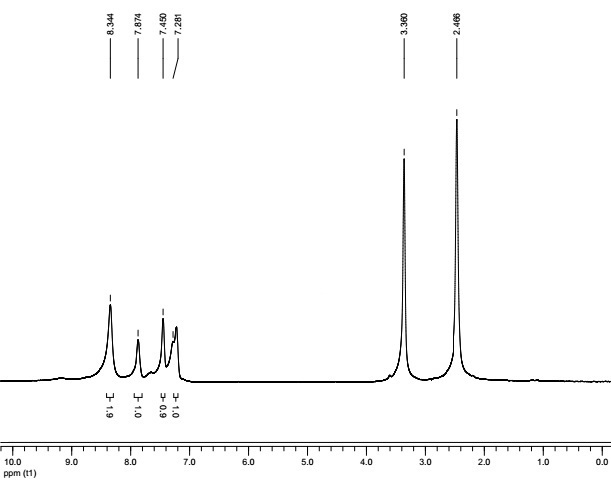
**

**^13^C NMR Spectrum**


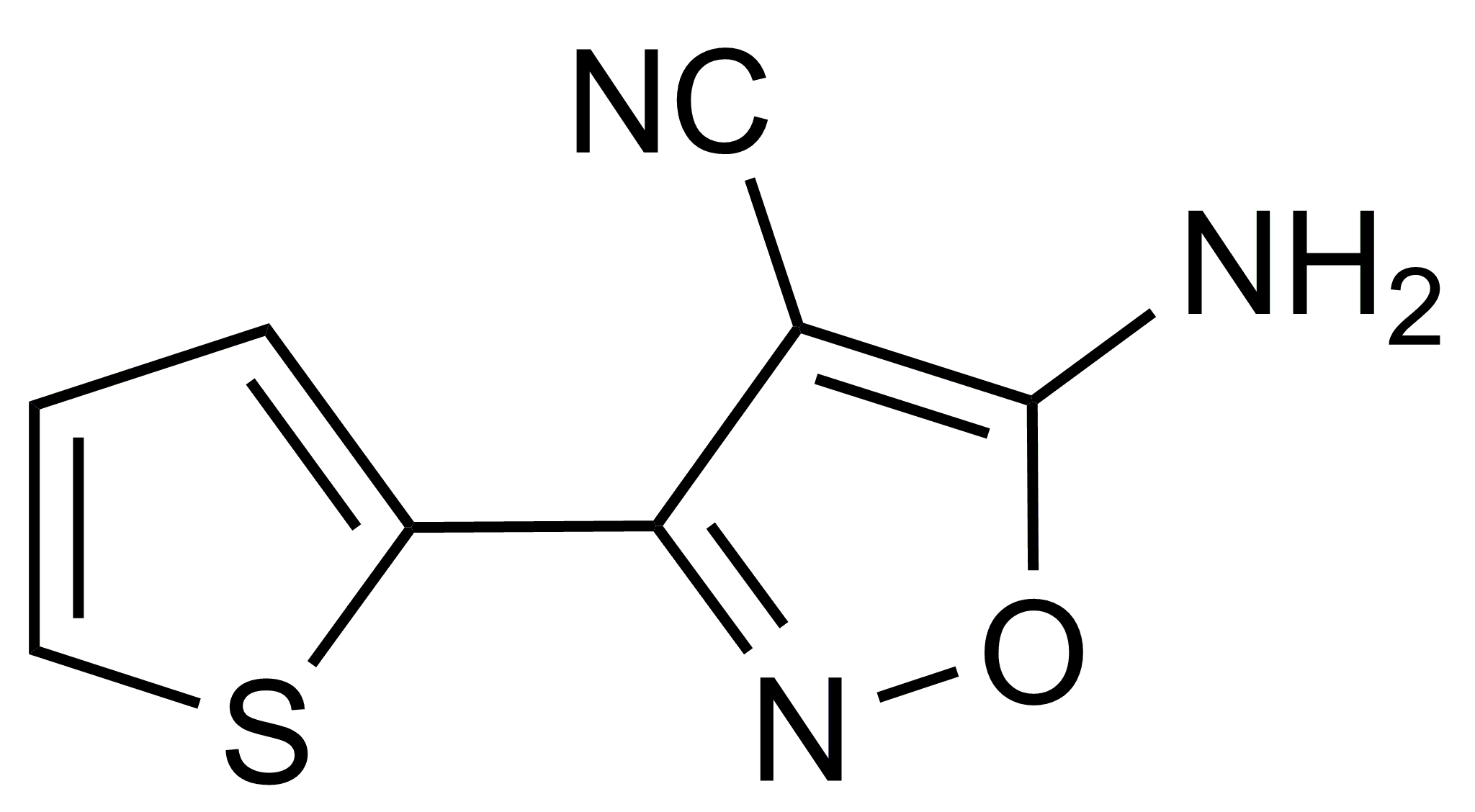

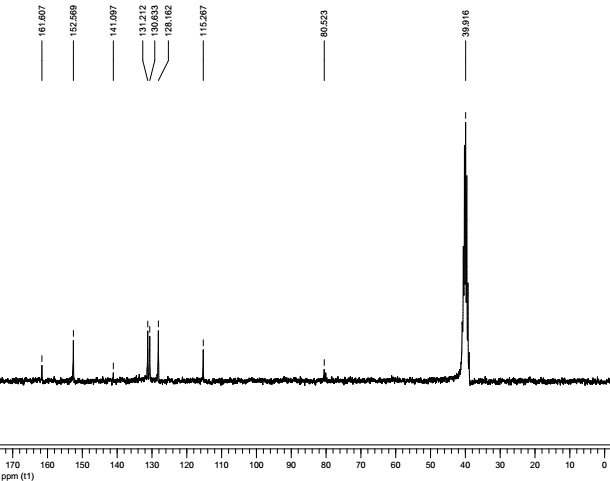


**9. Spectra of compound 4i**

**^1^H-NMR Spectrum**

**
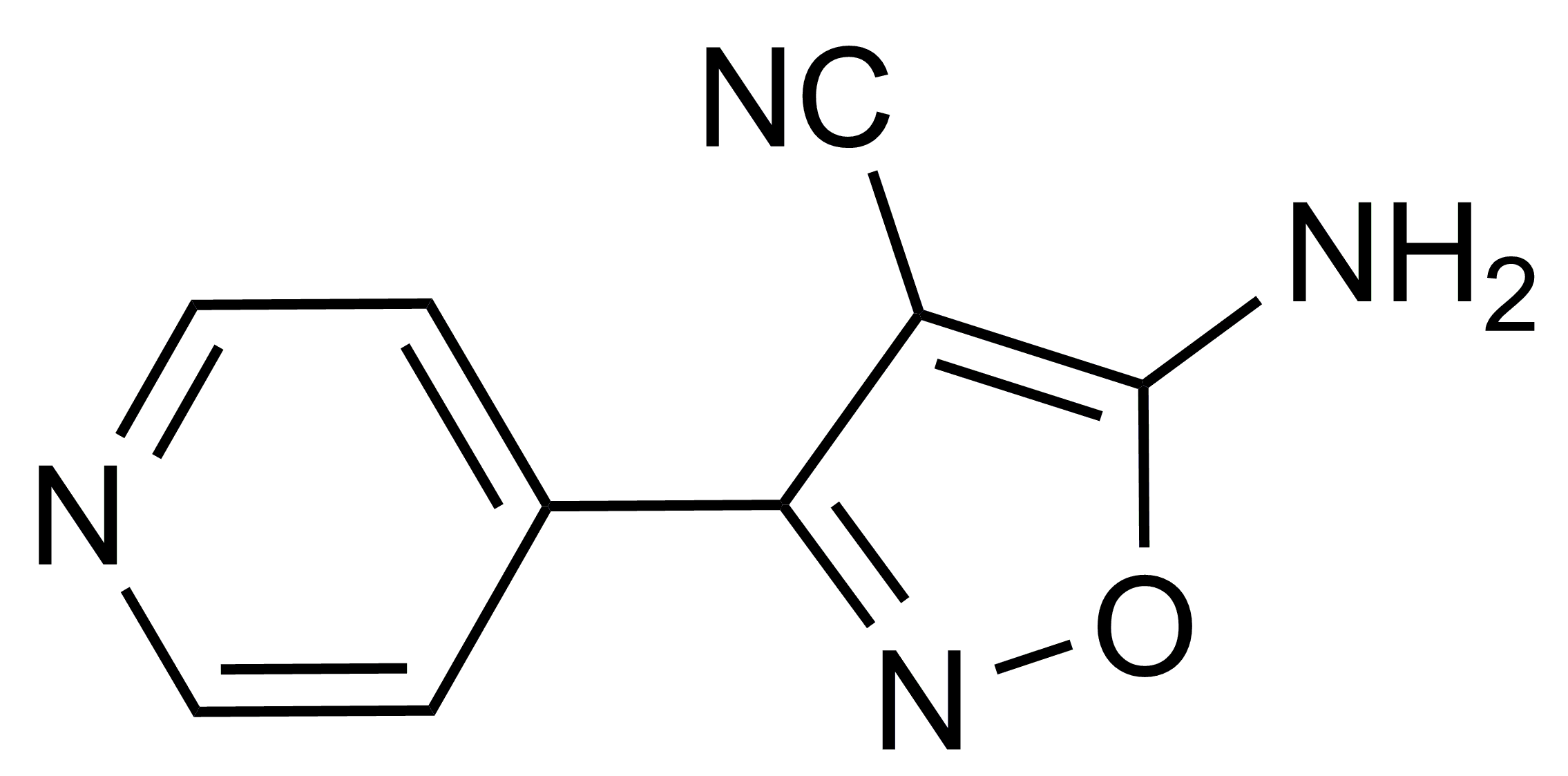

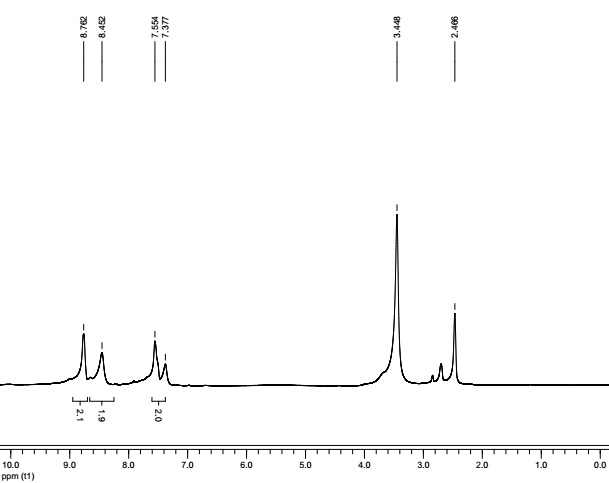
**

**^13^C NMR Spectrum**


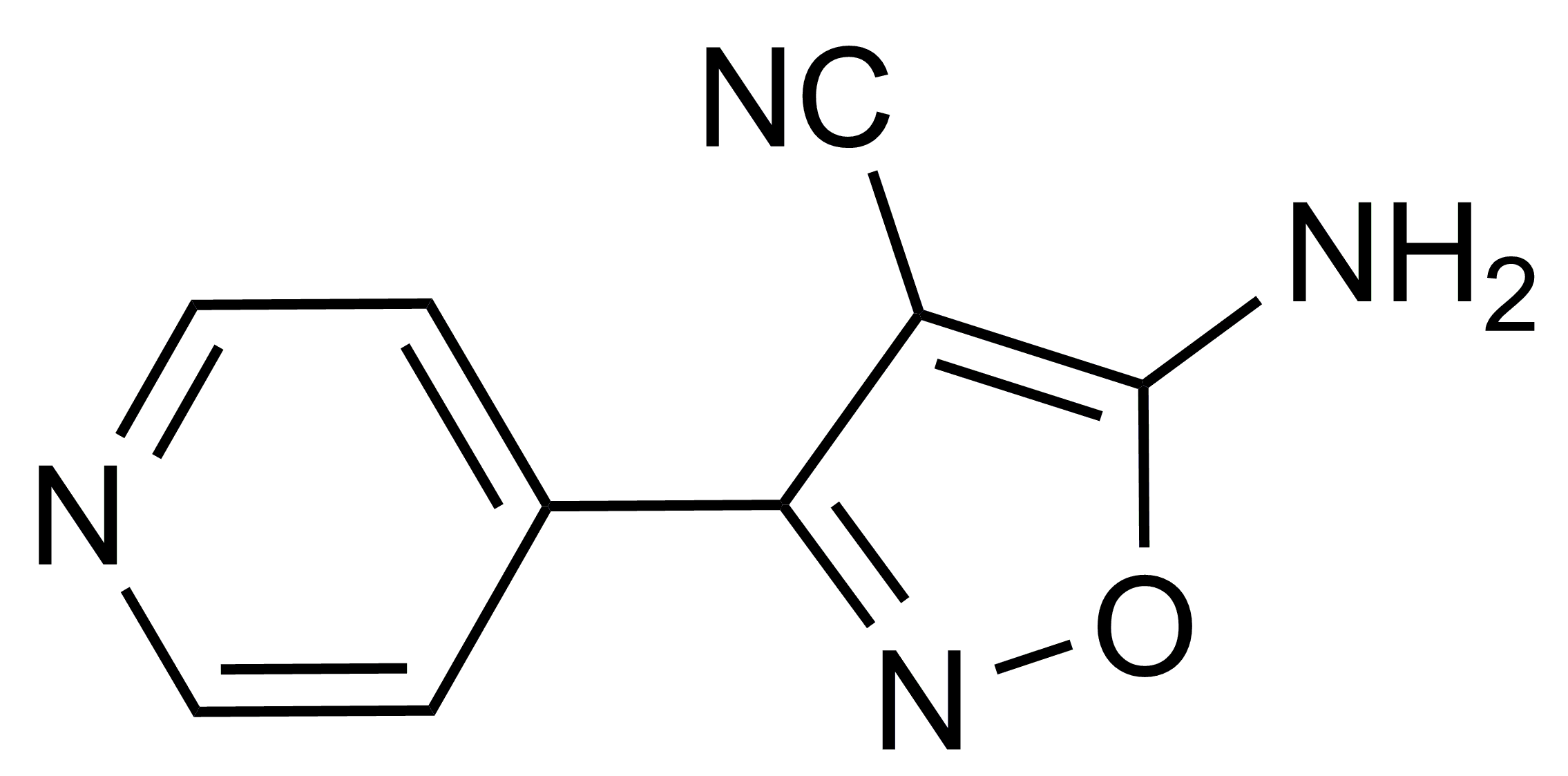

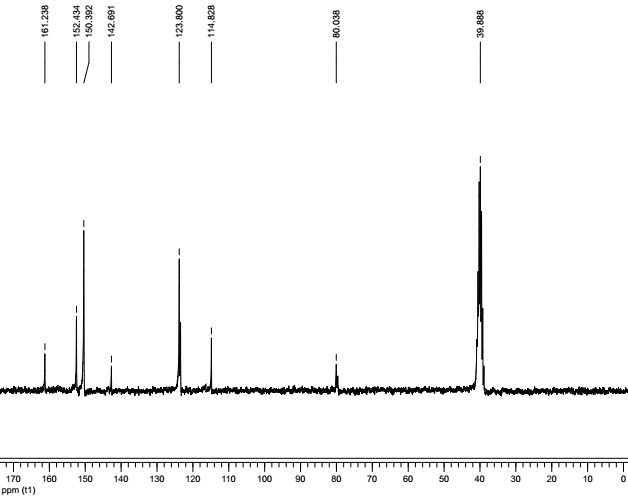

Supplement: Supplementary file 1 — Additional file 1. The copies of 1H NMR and 13C NMR spectra for isoxazoles 4a–i. [file 13065_2018_488_MOESM1_ESM.doc]
